# Exendin-4 alleviates steatosis in an in vitro cell model by lowering FABP1 and FOXA1 expression via the Wnt/-catenin signaling pathway

**DOI:** 10.1038/s41598-022-06143-5

**Published:** 2022-02-09

**Authors:** Olfa Khalifa, Neyla S. AL-Akl, Khaoula Errafii, Abdelilah Arredouani

**Affiliations:** 1 Diabetes Research Center, Qatar Biomedical Research Institute, Hamad Bin Khalifa University, Qatar Foundation, PO Box: 34110, Doha, Qatar; 2 College of Health and Life Sciences, Hamad Bin Khalifa University, Qatar Foundation, Doha, Qatar

**Keywords:** Cell biology, Molecular biology, Diseases

## Abstract

Non-alcoholic fatty liver disease (NAFLD) is the leading chronic liver disease worldwide. Agonists of the glucagon-like peptide-1 receptor (GLP-1R), currently approved to treat type 2 diabetes, hold promise to improve steatosis and even steatohepatitis. However, due to their pleiotropic effects, the mechanisms underlying their protective effect on NAFLD remain elusive. We aimed to investigate these mechanisms using an in vitro model of steatosis treated with the GLP-1R agonist Exendin-4 (Ex-4). We established steatotic HepG2 cells by incubating the cells with 400 µM oleic acid (OA) overnight. Further treatment with 200 nM Ex-4 for 3 h significantly reduced the OA-induced lipid accumulation (p < 0.05). Concomitantly, Ex-4 substantially reduced the expression levels of Fatty Acid-Binding Protein 1 (FABP1) and its primary activator, Forkhead box protein A1 (FOXA1). Interestingly, the silencing of β-catenin with siRNA abolished the effect of Ex-4 on these genes, suggesting dependency on the Wnt/β-catenin pathway. Additionally, after β-catenin silencing, OA treatment significantly increased the expression of nuclear transcription factors SREBP-1 and TCF4, whereas Ex-4 significantly decreased this upregulation. Our findings suggest that direct activation of GLP-1R by Ex-4 reduces OA-induced steatosis in HepG2 cells by reducing fatty acid uptake and transport via FABP1 downregulation.

## Introduction

Non-alcoholic fatty liver disease (NAFLD), defined as the excessive accumulation of lipids in the liver, is the most common cause of chronic liver disease in industrialized nations^1^ and the most frequent indication for liver transplantation^2,3^. NAFLD refers to a group of liver diseases that includes simple steatosis (benign fatty infiltration), non-alcoholic steatohepatitis (NASH) (fatty infiltration plus inflammation), fibrosis, and cirrhosis, which occasionally progresses to hepatocellular carcinoma^4^. NAFLD is associated with several comorbidities, including type 2 diabetes (T2D), cardiovascular diseases (CVD), and chronic kidney disease (CKD)^5^. The mechanisms underlying the above associations remain elusive. However, given the liver's crucial role in many aspects of the metabolism of lipids, carbohydrates, and proteins, it is appreciated that any injury to the liver will potentially impact several organs^6^. NAFLD's etiology is not fully elucidated. However, it is accepted that visceral adiposity, insulin resistance, T2D, hypertension, and dyslipidemia are significant contributors to NAFLD development^7^.

There is currently no approved pharmacotherapy for NAFLD. Hitherto, weight loss is the only intervention proven to be significantly beneficial for NAFLD patients^8^. Losing 5% of one's bodyweight improves abnormal liver tests and reduces liver fat^9^, whereas losing 7 to 10% of one's body weight appears to reduce inflammation and injury to liver cells and may even reverse some fibrosis damage^10^. Unfortunately, most people find it difficult to lose the weight they need to improve NAFLD and much more challenging to keep it off. Hence, there is an urgent need for novel therapeutic approaches to improve NAFLD independently of weight loss.

Agonists of the glucagon-like peptide-1 receptor (GLP-1R) have recently been investigated to treat NAFLD due to their bodyweight-lowering effects^11^. GLP-1 is a multifaceted hormone secreted by the L cells of the intestine^12^. Among other things, GLP1 regulates blood glucose levels by stimulating glucose-dependent insulin release and decreasing glucagon secretion, promotes proliferation of pancreatic b-cells, slows gastric emptying, and inhibits satiety and food intake through effects on central nervous system centers^13,14^. This pleiotropic effect is due to the expression of the GLP-1 receptor by various organs such as the pancreas, brain, kidney, gut, lung, heart, muscle, and liver^15^. Some GLP-1R agonists, like Liraglutide (taken once daily) or Dulaglutide (taken once weekly), are already licensed for T2D and obesity management in humans due to their ability to mimic the effects of GLP-1^16–18^.

Given their weight loss-inducing effect, by reducing satiety and food intake, the impact of the GLP-1R agonists on liver fat content has been investigated in numerous in vivo studies and yielded promising results^19–35^. As a result, these drugs are suggested as potential options for treating and slowing the progression of NAFLD. Nonetheless, it is unclear whether the protective effect of GLP-1R agonists on fat content stems from weight loss, which, among other things, increases insulin sensitivity and improves glycemia and lipid profile, or from direct activation of the hepatic GLP-1R. Gupta and colleagues^36^ were the first to report GLP-1 receptor expression in human hepatocytes and proposed that they play a direct role in reducing hepatic steatosis in vitro through the modulation of effectors of the insulin signaling pathway. Recently, Seo and coworkers^37^ suggested that the GLP-1R agonist Exendin-4 (Ex-4) reduces fat content in an in vitro cell model of steatosis by inhibiting hepatic lipogenesis through activation of β-catenin signaling and modulation of the expression of several lipogenesis genes. β-catenin was also suggested to mediate the effect of GLP-1 receptor agonist Exenatide on ameliorating hepatic steatosis induced by a high fructose diet in rats^38^. The β-catenin is an intracellular signal transducer in the Wnt signaling pathway, which maintains hepatic homeostasis and contributes to specific hepatic characteristics, including liver metabolism^39^ and metabolic zonation regeneration^40^.

Hepatic lipid content and homeostasis are determined by: (a) circulating free fatty acid uptake, (b) hepatic de novo lipogenesis, (c) hepatic β-oxidation, and (d) hepatic lipid export via very-low-density lipoprotein (VLDL)^41,42^. We used HepG2 cells treated with Oleic Acid (OA) as a model of hepatic steatosis in this study to see if direct activation of the GLP-1R with Ex-4 affects any of the four processes listed above and thus improves steatosis. The use of the in vitro model allows us to demonstrate the direct effect of Ex-4 on GLP-1R and to overcome the pleiotropic effect of GLP-1R agonism in vivo.

## Materials and methods

### HepG2 culture

We obtained the human hepatoma HepG2 cell line (HB-8065, ATCC) from ATCC (Manassa, Virginia, USA) and maintained it in Dulbecco's modified Eagle's medium (DMEM) (31966047, Gibco, Massachusetts, USA) at 37 °C and 5% CO2. DMEM was supplemented with 10% FBS (10500064, Gibco, Massachusetts, USA) and 1% penicillin/streptomycin (15070063, Gibco, Massachusetts, USA). We performed all the experiments with cells passaged fewer than 25 times.

### Preparation of oleic acid

We prepared the oleic acid solution as in^43^. Briefly, we dissolved the powder OA (O-1008 Sigma-Aldrich, Germany) at a final concentration of 12 mM in phosphate-buffered saline (PBS; 137 mM NaCl, 10 mM phosphate, 2.7 mM KCl, and pH 7.4) that contained 11% fatty acid-free bovine serum albumin (FFA-BSA; 0215240110, MP Biomedicals, Santa Ana, CA, USA). The solution was then sonicated and shaken at 37 °C overnight using an OM10 Orbital Shaking Incubator (Ratek Instruments Pty, Ltd., Boronia, Australia). The OA solution was filtered using a 0.22 µm filter, aliquoted, and stored at 4 °C. We used a fresh aliquot for each experiment.

### Induction of steatosis

**Figure 1:**
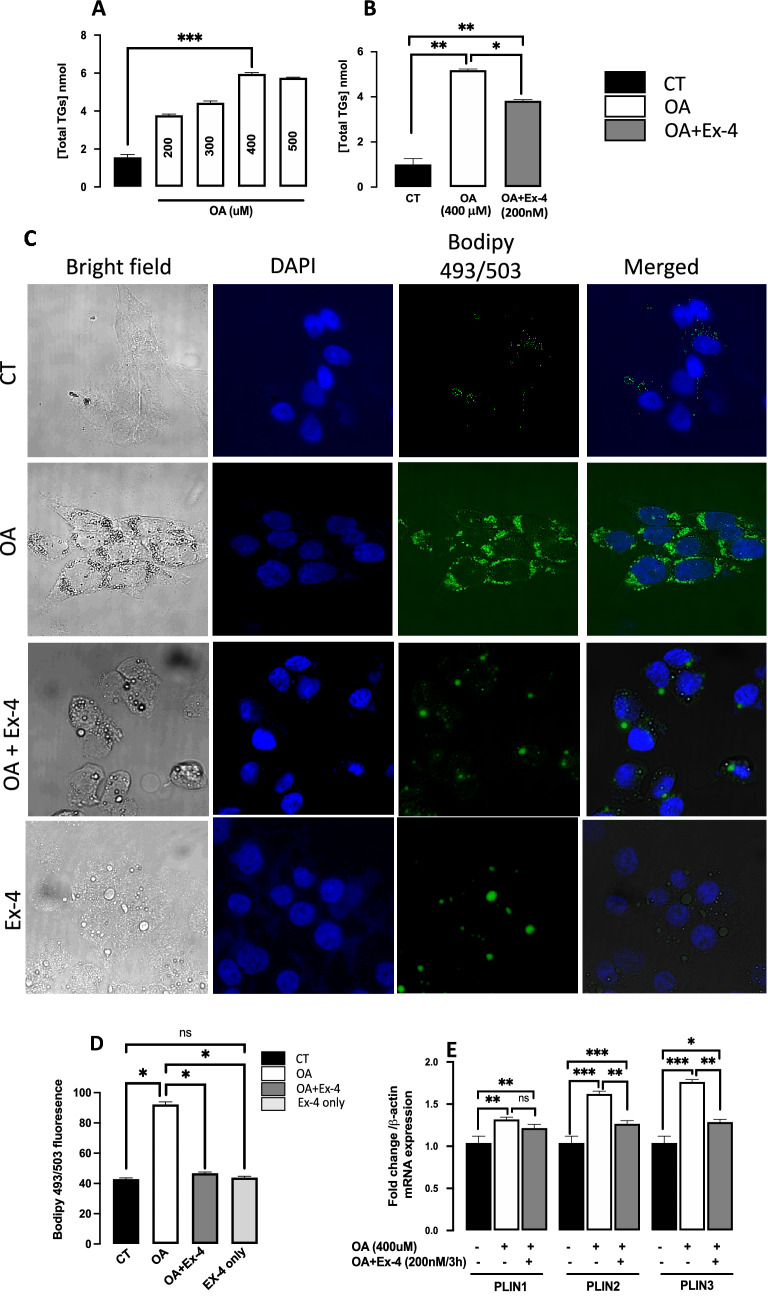
Exendin-4 reduces Oleic acid-induced lipid accumulation in HepG2 cells. Except from the experiment in panel (**A**), in all other experiments, HepG2 cells were starved for 6 h and then treated with Oleic acid (OA; 400 μM) for 16 h followed by 3 h treatment with OA with or without Exendin-4 (Ex-4; 200 nM). (**A**) OA dose-dependent TGs accumulation in HepG2 cells. (**B**) Exendin-4 significantly reduces OA-induced rise in TGs content in HepG2 cells. (**C**) Confocal imaging of lipid droplets after staining with 0.2 mM BODIPY 493/503 (green) and DAPI (blue). The white dots in the bright field images indicate lipid droplets (**D**) Quantification of the lipid content with the BODIPY/DAPI fluorescence ratio in the presence of OA or OA + EX-4. We analyzed 200 cells for each condition. (**E**) Quantification of mRNA expression levels of perilipin 1 (Plin1), perilipin 2 (Plin2) and perilipin 3 (Plin3). The expression levels were normalized to the level of β-actin. All values are expressed as the mean ± SE (n = 6). *p < 0.05, **p < 0.01, ***p < 0.001.

To establish the steatosis cell model, we first determined the optimal concentration of OA needed to obtain saturating levels of triglycerides (TGs). To this aim, we cultured HepG2 cells in 6-well plates at a density of 4 × 10^5^ cells/well until 70% confluence. We then starved the cells for 6 h in DMEM containing 1% fatty-acid-free bovine serum albumin. Following the starvation, a 16-h incubation in DMEM containing increasing concentrations of OA (0–500 µM) at 37 °C was performed, and steatosis was quantified (Fig. 1A).

### Treatment with exendin-4

After steatosis induction, the cells were washed and incubated in fresh DMEM containing 400 μM OA in the absence or presence of Ex-4 (E7144-0.1MG, Tocris, Minneapolis, Minnesota). To determine the optimal concentration of Ex-4, we treated the steatotic cells with increasing concentrations of Ex-4 from 0 to 1 mM and with different incubation periods (3, 6, 12, and 24 h). We then quantified the TG content as above. We used a fresh aliquot of EX-4 for each experiment.

**Table 1:** Primer list and sequences.

Gene	GenBank IDs	Forward sequence (5′ 3')	Reverse sequence (5′ 3')	PCR product sizes (pb)
SREBP-1	U00968.1	GGCTCCTGCCTACAGCTTCT	CAGCCAGTGGATCACCACA	109
PPARγ	AB247367.1	GACCTCAGACAGATTGTCAC	AGTCCTTGTAGATCTCCTGC	106
SCD1	NM_005063.5	CACCACATTCTTCATTGATTGCA	ATGGCGGCCTTGGAGACT	75
FAS	BC063242.1	TATGCTTCTTCGTGCAGCAGTT	GCTGCCACACGCTCCTCTAG	94
ACC	NM_198838.2	CAGAAGTGACAGACTACAGG	ATCCATGGCTTCCAGGAGTA	125
DGAT1	NM_012079.6	AACTGGTGTGTGGTGATGCT	CCTTCAGGAACAGAGAAACC	112
DGAT2	AY358532.1	CTACAGGTCATCTCAGTGCT	GAAGTAGAGCACAGCGATGA	120
β-catenin	NM_001330729.2	GCAAGCTCATCATACTGGCT	CTTGCATTCCACCAGCTTCT	162
PLIN1	NM_001145311.2	GATCATGAGGACCAGACAGA	CTGCTACCTCACTGAACTTG	91
PLIN2	NM_001122.4	ACAGACCATTTCTCAGCTCCAT	TATCCAATGCTCCTTTTCCACT	141
PLIN3	NM_001164194.2	GAACAGAGCTACTTCGTACG	CAGTTTCCATCAGGCTTAGG	151
FOXA1	NM_004496.5	GCAATACTCGCCTTACGGCT	TACACACCTTGGTAGTACGCC	128
ApoB	NM_000384.3	TGCTCCACTCACTTTACCGTC	TAGCGTCCAGTGTGTACTGAC	199
FABP1	NM_001443.3	ATGAGTTTCTCCGGCAAGTAC	CTCTTCCGGCAGACCGATT	81
GLP-1R	2740	TTG GGG TGA ACT TCC TCA TC	CTT GGC AAG TCT GCA TTT GA	74
β-actin	NM_001101.5	TCATGAAGATCCTCACCGAG	CATCTCTTGCTCGAAGTCCA	116

### Quantification of steatosis

We used three methods to quantify steatosis in HepG2 cells:

1. Quantification of triglycerides
  We measured total TGs levels using a commercial fluorometric assay kit (Abcam TG quantification assay kit, ab65336) and a microplate reader (Infinite F200 Pro; Tecan, Switzerland). The kit converts triglycerides to free fatty acids and glycerol. Glycerol is then oxidized to generate a product that reacts with a probe to generate fluorescence when excited at 535 nm. The emitted fluorescence is collected at 587 nm. We calculated the TGs content from a standard curve prepared for each assay using known TGs concentrations. We normalized the data to total cellular protein content.
2. Staining of neutral lipids with BODIPY 493/503
  To visualize the accumulation of lipids in response to OA treatment, we used boron-dipyrromethene (BODIPY) 493/503 (D3922, Thermo Fisher Scientific, MA, USA), which labels specifically intracellular neutral lipids^44^. Briefly, we grew HepG2 on 12 mm coverslips until 70% confluence, starved them, and then treated them with OA and Ex-4 as needed. After a quick wash, we fixed the cells with 4% paraformaldehyde for 7 min, washed them with PBS, and then incubated them for 10 min with 0.2 μM BODIPY 493/503. We further labeled the nuclei by incubating the cells with 1 μM DAPI for 1 min. After a final wash with PBS, we mounted the coverslips on microscope slides used for imaging on a Zeiss LSM 870 confocal microscope, as we reported recently^45^. To analyze the images, we used ImageJ software (version 1.8.0, NIH, USA). The intracellular lipid accumulation was calculated by dividing the BODIPY fluorescence intensity by that of DAPI. Two independent researchers analyzed 200 individual cells for each condition (untreated, steatotic, and Ex-4-treated steatotic cells) from three different experiments.
3. Relative expression of perilipin genes
  Perilipin family proteins, with five recognized members (PLIN1-5), are found on the surfaces of intracellular lipid droplets^46^. We used qRT-PCR to quantify the relative expression of PLIN1, 2, and 3 and estimate the lipid accumulation in response to OA and EX-4 treatments. The primers we utilized for the genes are listed in Table 1.

### Quantification of lipogenesis gene expression

To quantify gene expression, we used the Pure Link RNA Mini kit (12183025, Invitrogen, USA), Hilden, Germany) to extract total RNA from untreated and treated HepG2 cells and used High-Capacity cDNA Reverse Transcription kit (4368813, Applied Biosystems, Foster City, CA, USA) and 2 μg total RNA to prepare cDNA. We quantified gene expression by qRT-PCR on QuantStudio 6 Flex system (ThermoFisher, Waltham, MA), using PowerUp™ SYBR™ Green Master Mix (A25780, Applied Biosystems, USA). We normalized the data to β-actin as an internal control and used the comparative 2^-ΔΔCT^ method to calculate the relative expression. We have quantified the expression level of the following genes: Fatty Acid Synthase (FAS), Acyl-CoA Dehydrogenase Long Chain (ACADL), Carnitine Palmitoyltransferase 1A (CPT1A), Stearoyl-CoA Desaturase 1 (SCD-1), Acetyl-CoA Carboxylase Alpha (ACC), Diacylglycerol O-acyltransferase 1 (DGAT1), Diacylglycerol O-acyltransferase 2 (DGAT2), Sterol Regulatory Element Binding Transcription Factor 1 (SREBP-1), Peroxisome Proliferator-Activated Receptor Gamma (PPARγ), Fatty Acid Binding Protein 1 (FABP1), Forkhead box A1 (FOXA1) and Apolipoprotein B (APOB). Table 1 lists the sequences of the primers we used in this study. We used Primer-BLAST (https://www.ncbi.nlm.nih.gov/tools/primer-blast/) to design specific primers that met the following criteria: (1) Primer pairs are unique. They will not bind to other locations in the genome except the intended gene or DNA fragment. (2) Primer pairs do not bind to each other (forming primer dimer): self-or hetero-dimer. (3) The possibility of forming the secondary structure of the primers, which may cause difficulties for PCR amplification, is very low. (4). Tm (temperature of mismatch) of two primers is designed to be close to each other. (5) TA (Annealing temperature) is much lower than Tm. Moreover, our primers were analyzed by 'OligoAnalyzer 3.1' program from IDT company (http://www.idtdna.com/calc/analyzer).

### Gene silencing with siRNA

For siRNA-mediated β-catenin gene silencing, we transfected HepG2 cells with 5 nM of β-catenin-specific siRNA or Stealth siRNA negative control, obtained from Dharmacon (Lafayette, Colorado, USA), using Lipofectamine RNAiMAX transfection kit (13,778–075; Invitrogen, MA, USA) according to the manufacturer's instructions. After transfection, cells were cultured under normal growth conditions (37 °C, 5% CO2) for 24 h without antibiotics. The silencing efficiency was checked by quantifying the expression of β-catenin with qRT-PCR. For GLP-1R gene silencing, we used the Dicer-Substrate Short Interfering RNAs (DsiRNAs) and TriFECTa^®^ Kits (http://www.idtdna.com/calc/analyzer) and the Lipofectamine RNAiMAX transfection kit (13778-075; Invitrogen, MA, USA) to transfect HepG2 cells with 20 nM of GLP-1R specific siRNA or negative scrambled siRNA, according to the manufacturer's instructions. The DsiRNAs-TriFECTa^®^ kit contains three Dicer-substrate 27-mer RNA duplexes specific for a single target gene. A pool of the three duplexes was used to silence GLP-1R. After the silencing of GLP-1R, a qRT-PCR was performed for the following genes: PPARγ, FAS, DGAT1, DGAT2, and ACC. We normalized the data to β-actin as an internal control and used the comparative 2-ΔΔCT method to calculate the relative expression.

### Western blotting

After treating HepG2 cells with OA and Ex-4, we extracted nuclear and cytoplasmic proteins using the PARIS™ Kit (AM1921, Ambion® PARIS™, Invitrogen, MA, USA). We resolved 20 mg of proteins on 10% Tris–Glycine Mini Gels (Novex, XP00100BOX, Thermo Fisher Scientific) and then transferred them to a 0.2 mM polyvinylidene difluoride (PVDF) membrane using the Trans-Blot Turbo (Bio-Rad, California, USA). After the transfer, we incubated the membranes for 1 h at room temperature with the following primary antibodies: anti-β-catenin (#9582; Cell signaling, Danvers, MA, USA), anti-TCF4 (#2569; Cell signaling, Danvers, MA, USA), anti-SREBP-1 (sc-365513; Santa Cruz Biotechnology, Texas, USA), anti-β-actin (#4970; Cell signaling, Danvers, MA, USA), anti-Lamin B1 (ab16048; Abcam, MA, USA), anti-FOXA1 (ab23738; Abcam, MA, USA), anti-FABP1 (13368S, Cell Signaling, Ma, USA and anti-ACC (3676S, cell signalling, MA, USA) . After 3 × 10 min washes with PBS, we incubated the membranes with the appropriate horseradish peroxidase-conjugated secondary antibody. We developed the membranes with the super signal west Femto Maximum Sensitivity Substrate (34094, Thermo Fisher Scientific, USA), and Immunoreactive bands were detected by chemiluminescence on Biorad ChemiDOC XRS (Biorad, CA, USA) machine. We normalized the results to β-actin as an internal control for total proteins and Lamin-B1 for nuclear proteins. We used the dilutions recommended by the manufacturer for all antibodies unless otherwise stated.

### Statistical analysis

We performed the statistical analysis and the graphing with GraphPad Prism 9.0 software (GraphPad Prism v9, La Jolla, CA, USA). Data are presented as the mean ± SEM. We used unpaired one-way ANOVA analysis (ANOVA) to assess the significance of differences in mean values between experimental groups, and Tukey's posthoc test was used to adjust multiple comparisons between experimental groups. When we silenced β-catenin*,* we used a two-way analysis of variance (ANOVA) to evaluate the significance of differences between the mean values of different experimental groups. Unless otherwise specified, a p-value of < 0.05 was considered significant.

## Results

### Exendin-4 reduces lipid content in OA-treated HepG2 cells

By treating HepG2 cells with increasing OA concentrations for 16 h and measuring TG accumulation, we determined the optimal concentration of OA required to induce steatosis (Fig. 1A). With 200 mM OA, we obtained a significant accumulation of TGs, but with 400 mM, we obtained saturating levels of TGs (p < 0.001, relative to untreated). As a result, we used 400 mM OA to induce steatosis in all our experiments. On the other hand, we found that treating steatotic cells with 200 nM Ex-4 for 3 h is optimal for reducing lipid accumulation significantly (data not shown). We then compared TGs content between untreated cells, steatotic cells, i.e., cells treated with OA alone (400 µM /16 h), and steatotic cells treated with Ex-4 (200 nM /3 h) in the continuous presence of 400 µM OA (OA + EX-4). Figure 1B shows that in the presence of Ex-4, the TGs content was significantly lower than OA alone (p < 0.05), suggesting that Ex-4 reduces the OA-induced lipid accumulation. Furthermore, confocal microscopy analysis of BODIPY-stained untreated, steatotic, and Ex-4-treated steatotic cells showed that Ex-4 significantly decreases the number of lipid droplets (Fig. 1C), confirming the significant reduction of the OA-induced accumulation of lipids (p < 0.01) (Fig. 1D). We have also looked at the effect of Ex-4 on BODIPY staining in the absence of OA and found that it is also significantly lower than OA alone (Fig. 1C,D).

PLIN proteins play a role in forming lipid droplets and regulating lipid storage^47^. PLIN4 is absent in the liver and expressed weakly in the heart and skeletal muscle^48^, whereas PLIN5 is expressed at a low level in the liver^49^. Previously, Carr and colleagues^50^ reported that PLIN1 and PLIN2 proteins are upregulated in hepatic steatosis and adult NASH. Since PLINs are associated with lipid droplets, their relative expression is proportional to the number of lipid droplets.

We quantified gene expression of the lipid droplet binding proteins PLIN1, 2, and 3 and found that OA significantly increases the expression of these genes (Fig. 1E), suggesting an increase in the number of lipid droplets. However, in the presence of Ex-4, the expression of PLIN2 and PLIN3, but not PLIN1, was significantly lower than OA alone, indicating that Ex-4 reduces the number of lipid droplets, and thus the lipid content.

### Exendin-4 counteracts the effect of OA on the expression of lipogenesis genes in HepG2 cells

**Figure 2:**
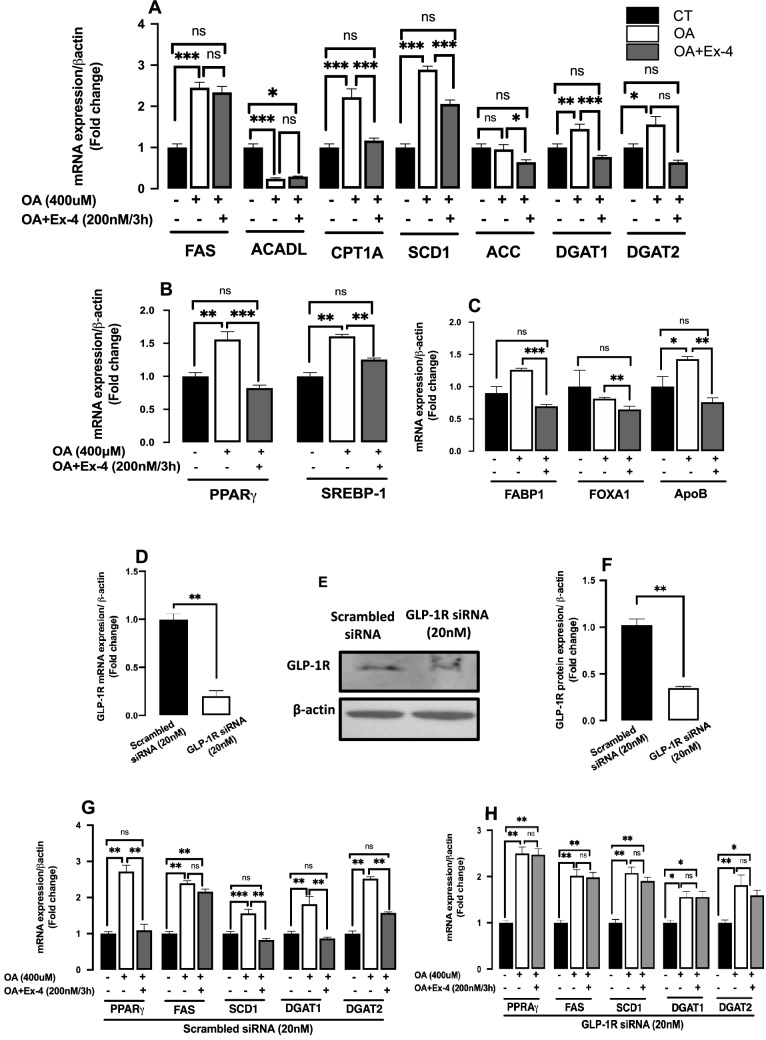
Exendin-4 affects hepatocyte lipid metabolism genes by stimulating the GLP-1R. HepG2 cells were starved for 6 h and then treated with Oleic acid (OA; 400 μM) for 16 h followed by 3 h treatment with OA with or without exendin-4 (Ex-4; 200 nM). The expression levels of different genes were quantified with qRT-PCR and normalized to the level of β-actin. (**A**–**C**) The mRNA expression levels of FAS (Fatty acid synthase), ACADL (acyl-CoA dehydrogenase long chain), CPT1A (carnitine palmitoyltransferase 1A), SCD1(stearoyl-CoA desaturase), ACC (acetyl-CoA carboxylase alpha), DGAT1 (diacylglycerol O-acyltransferase 1), DGAT2 (diacylglycerol O-acyltransferase 2), SREBP-1 (sterol regulatory element-binding transcription factor 1), PPARγ (peroxisome proliferator-activated receptor-gamma), FABP1(fatty acid-binding protein 1 ), FOXA1 (forkhead box A1), and APOB (apolipoprotein B) after treatment with OA alone or OA + Ex-4. (**D**) Silencing of GLP-1R. HepG2 cells were transfected with 20 nM siRNA directed against GLP-1R for 24 h , GLP-1R mRNA expression was quantified with qRTPCR. (**E**,**F**) GLP-1R proteins expression was quantified with western blot in HepG2. Full-length blots are displayed in Supplementary Fig.S.1. (**G**,**H**) mRNA expression levels of PPARγ, FAS, SCD1, DGAT1, and DGAT2 after transfection with scrambled GLP-1R siRNAs. All values are expressed as the mean ± SE (n = 6). * p < 0.05, ** p < 0.01, *** p < 0.001.

Compared to untreated HepG2 cells, steatotic cells showed a significant upregulation of the lipogenesis genes SREBP-1, PPARγ, FAS, CPT1A, SCD1, DGAT1, and DGAT2 (Fig. 2A,B), while ACADL expression was significantly downregulated and ACC expression was unaffected**.** Interestingly, when compared to OA alone, the presence of Ex-4 significantly decreased the expression of SREBP-1, PPARγ, CPT1A, ACC, DGAT1, and SCD1 while the expression of ACADL, DGAT2 and FAS remained unaffected (Fig. 2A,B). Furthermore, while OA treatment did not significantly change the expression levels of FABP1 and FOXA1 relative to untreated cells, Ex-4 treatment significantly reduced the expression of these genes compared to OA treatment alone (Fig. 2C). The ApoB expression, on the other hand, was significantly increased by OA treatment, but this increase was significantly reversed by Ex-4 treatment (Fig. 2C). We then looked into whether the Ex-4's impact on some of these genes is mediated via the GLP-1R. To that purpose, we used specific siRNA to silence the GLP-1R and then examined the expression of PPARγ, FAS, SCD1, DGAT1, and DGAT2 genes under the different treatment settings. As illustrated in Fig. 2D–F, we achieved about 70% (p < 0.01) and 65% (p < 0.01) GLP-1R silencing at the mRNA and protein levels, respectively. Furthermore, whereas GLP-1R silencing did not affect gene expression in the presence of OA alone, we observed that the effect of Ex-4 on gene expression with scrambled siRNA is reversed by GLP-1R silencing (Fig. 2G,H), indicating that GLP-1R is required for the action of Ex-4.

### Exendin-4 activates the β-catenin pathway in HepG2 steatotic cells

**Figure 3:**
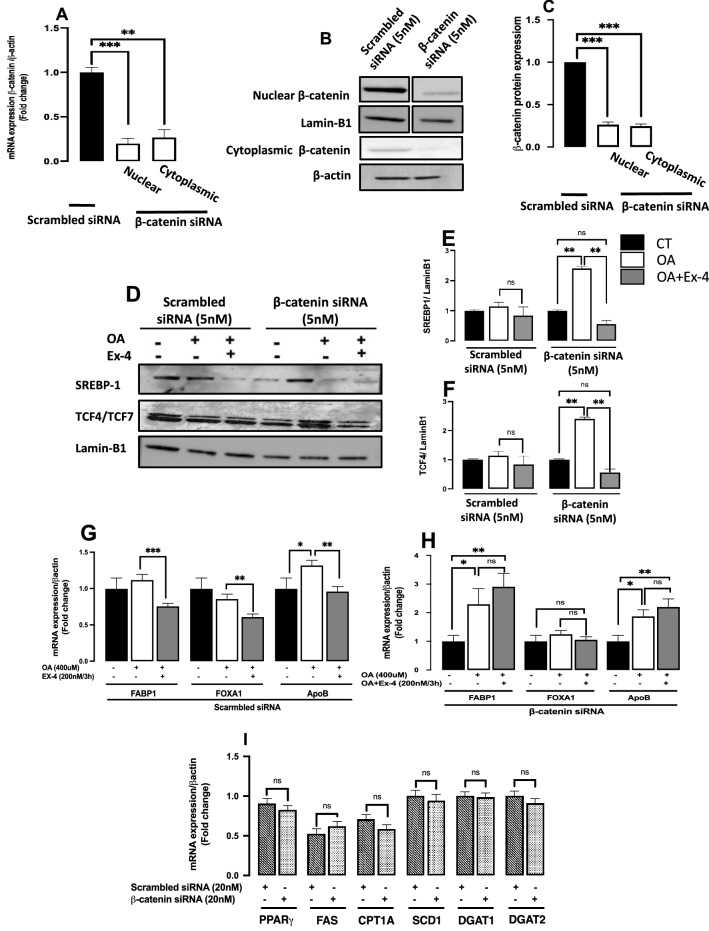
Ex-4 downregulates the expression of lipogenic transcription factors via the β-catenin pathway. Cytosolic and nuclear extracts were prepared from HepG2 cells transfected with 5 nM siRNA directed against β-catenin for 24 h and then treated with 400uM OA in the absence or presence of 200 nM Ex-4. (**A**–**C**) Silencing and quantification of β-catenin expression in cytoplasmic and nuclear fractions Full-length blots are displayed in Supplementary Fig.S.2 and S.3. (**D–F**) western blotting and quantification of the transcription factors SREBP-1 and TCF4. Nuclear proteins were normalized against Lamin-B1. All values are expressed as the mean ± SE (n = 6). * p < 0.05, ** p < 0.01, *** p < 0.001. Full-length blots are displayed in Supplementary Fig.S.4.

Seo and colleagues^37^ previously reported the activation of the β-catenin pathway in response to Ex-4. Here we confirm this activation by silencing the β-catenin with siRNA and testing the effect of Ex-4 on the expression of the nuclear factors SREPB-1 and TCF4, master transcription factors involved in the Wnt/β-catenin signaling. The knockdown efficiency at the mRNA level was 70% and 65% for the cytoplasmic and nuclear fractions, respectively (Fig. 3A). Similar results were obtained at the protein level (Fig. 3B,C). After silencing β-catenin, the significant OA-induced upregulation of both SREPB-1 and TCF4 was reversed by Ex-4 (Fig. 3D–F), indicating the involvement of the β-catenin pathway in the effect if Ex-4.

### Exendin-4 reduces FABP1 and FOXA1 expression through the activation of β-catenin signaling

To better understand the potential role of β-catenin as a molecular determinant through which Ex-4 mediates its beneficial effect on steatosis, we quantified the expression of FABP1, FOXA1, and ApoB after β-catenin silencing. Compared to the scrambled siRNA transfection (Fig. 3G), the OA significantly increased FABP1 mRNA expression, relative to untreated cells, following β-catenin knockdown (Fig. 3H, p = 0.032). However, the effect of OA on FOXA1 and ApoB expression, relative to untreated cells, was comparable between scrambled transfection and by β-catenin knockdown (Fig. 3G,H). Interestingly, Ex-4 significantly reduces the expression of FABP1, FOXA1, and ApoB, relative to OA alone, after scrambled transfection (Fig. 3G), but this downregulation is reversed after β-catenin knockdown, (Fig. 3H). We then looked into the effect of β-catenin silencing on the expression of PPARγ, FAS, CPT1A, SCD1, DGAT1, and DGAT2 mRNAs and found no significant effect (Fig. 3I).

**Figure 4:**
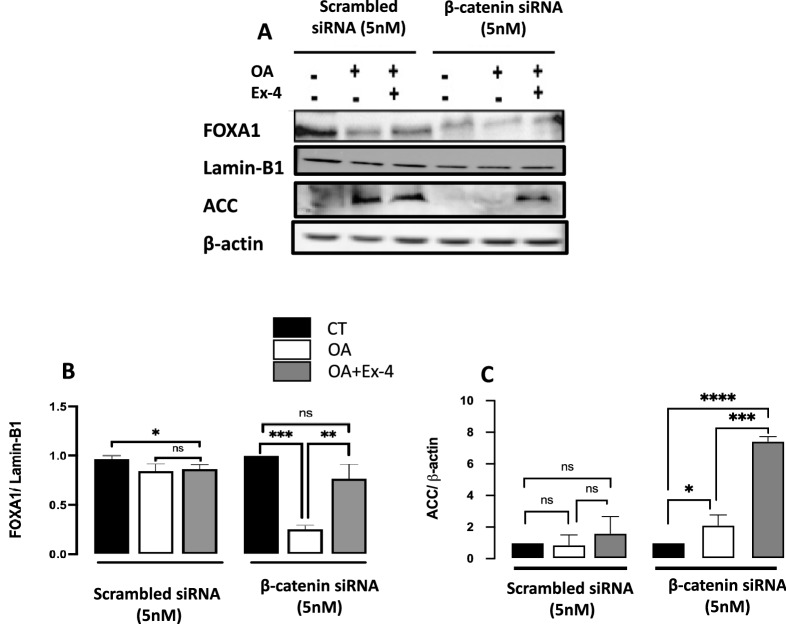
Effect of EX-4 on the protein expression of FOXA1 and ACC implicates β-catenin pathways. (**A**) Expression of FOXA1 and ACC was analyzed by western blotting in HepG2 cells transfected with 5 nM scrambled siRNA or β-catenin specific siRNA and then treated with 400 mM OA or 400 mM OA + 200 nM Ex-4 as indicated. (**B,C**) Quantification of the blots in (**A**). For ACC, beta-actin was employed as a loading control, while Lamin-B1 was used for FOXA1. All values are expressed as the mean ± SE (n = 3). * p < 0.05, ** p < 0.01, *** p < 0.001. Full-length blots are displayed in Supplementary Fig.S.5.

We further tested the effect of β-catenin silencing on the expression of FABP1, FOXA1 at the protein level (Fig. 4A–C). We could not detect FABP1 with the antibody we used, despite using up to 60 mg of protein and 1/200 antibody dilution (the company recommends 1/1000 dilution). Unlike the mRNA expression levels (Fig. 3H), OA significantly downregulated the level of FOXA1 protein level following β-catenin silencing (Fig. 4B). This downregulation was significantly reversed with Ex-4 (Fig. 4B). Together, these observations suggest a posttranslational regulation that implicates the β-catenin pathway. Furthermore, we tested the effect of β-catenin silencing on ACC. ACC catalyzes the ATP-dependent carboxylation of acetyl-CoA to malonyl-CoA in a multistep reaction. It's the first committed step in fatty acid synthesis, is rate-limiting for the pathway, and is tightly regulated. As shown in Fig. 4A,C, after β-catenin silencing, OA significantly increases the expression of ACC at the protein level, and Ex-4 further enhances this increase. The impact of Ex-4 on the the AC protein level contracts with its impact on the mRNA, suggesting a posttranslational regulation that implicates the β-catenin pathway.

## Discussion

In this study, we investigated the possible mechanisms underlying the protective effect of the GLP-1R agonist Ex-4 on hepatic steatosis in an in vitro cell model. We used the HepG2 cell line treated with oleic acid as a steatosis model and confirmed that Ex-4 significantly reduces OA-induced lipid accumulation. GLP-1R agonists have a wide range of complex physiological effects due to the widespread expression of the GLP-1 receptors throughout the body^14^. Because of this pleiotropic effect, distinguishing between direct, i.e., via agonist-receptor interaction, and indirect effects of these agonists in vivo is challenging. Therefore, it remains unclear whether the reduction of steatosis observed in animal and human trials in response to treatment with GLP-1R agonists results from direct activation of hepatic GLP-1R or the indirect impact such as weight loss, increased insulin sensitivity, brain-liver signals such as brain leptin^51^, or other hormonal signals that these agonists might trigger^14^. To overcome this challenge, we opted for the in vitro model to ascertain that Ex-4's effect on steatosis results from direct activation of the GLP-1R.

We found that the effect of Ex-4 on different lipid metabolism genes is abrogated following the silencing of the GLP-1R (Fig. 2G,H), indicating that Ex-4's effect is mediated through GLP-1R. The most important finding of our study is the significantly lower expression of FABP1 (also known as liver-type fatty acid-binding protein or L-FABP) in Ex-4-treated cells compared to steatotic cells (Fig. 2C). Fatty acid‐binding proteins (FABPs) are small cytoplasmic proteins involved in intracellular lipid metabolisms such as fatty acid uptake, transport to mitochondria or peroxisome for oxidation, lipid synthesis, storage in lipid droplets, and regulation of nuclear receptors^52^. FABP1 is highly expressed in hepatocytes and is required for FFA uptake and shuttling^53^. Previously, Wolfrum and coworkers^54^ elegantly showed that increasing the FABP1 expression by treating HepG2 cells with the potent peroxisome proliferators bezafibrate and Pirinixic acid leads to increased uptake of radio-labeled oleic acid by 38% and 78%, respectively. Conversely, decreasing FABP1 expression by antisense FABP1 mRNA to one-sixth of its regular expression reduces the ratio-labeled oleic acid uptake rate by 66%. Similar results were obtained in FABP1^–/–^ mice following intravenous bolus administration of OA^55^. These findings indicate a direct correlation between FABP1 expression and fatty acid uptake in the liver.

The Ex-4-induced FABP1 downregulation correlates with the significant reduction in TGs content observed under the same treatment (Fig. 2C). Interestingly, the silencing of β-catenin with siRNA abrogates the effect of Ex-4 on FABP1 expression (Fig. 3G,H), indicating its dependency on β-catenin signaling. To our knowledge, this is the first time a reduced FABP1 expression in response to direct activation of the GLP-1R is shown in hepatocytes. Previously, Panjwani and colleagues reported significantly reduced levels of TGs and FABP1 in liver cells from high-fat diet-fed male ApoE^(-/-)^ mice treated with taspoglutide, a long-lasting GLP-1R agonist^56^. However, the authors suggested the effect of taspoglutide was indirect as they could detect neither the protein nor the mRNA of GLP-1R in liver cells. However, it is worth noting that several studies have reported GLP-1R expression in both human and rodent hepatocytes^36,57^. We have also detected GLP-1R expression in HepG2 cells by western blotting and quantitative PCR (data not shown). Additionally, a recent study investigating the effect of the GLP-1R agonist liraglutide on obesity-induced chronic kidney injury in obese rats showed that the agonist significantly reduced the lipid content and, concomitantly, the expression level of FABP1 protein in the obese kidney, relative to untreated rats^58^.

In principle, four separate mechanisms may lead to hepatic lipid accumulation: (a) enhanced uptake of circulating free fatty acids, (b) increased hepatic de novo lipogenesis, (c) diminished hepatic β-oxidation, and (d) decreased hepatic lipid export via VLDL^41,42^. Therefore, one explanation for the Ex-4-induced improvement in steatosis observed in our model could be a decreased fatty acid uptake by FABP1. This explanation is consistent with the fact that FABP1 silencing in mice reduces liver weight and hepatic TG content^59,60^, whereas FABP1 overexpression increases hepatic fatty acid uptake^61^. Moreover, the expression of FABP1 is significantly higher in the liver in obese patients with simple steatosis than in the obese healthy group^62^.

We have also observed that the presence of Ex-4 decreases the expression of ACC and DGAT1 (Fig. 2A), which are critical rate‐limiting enzymes for fatty acid biosynthesis and TG formation, respectively^63,64^. Previous research on DGAT1^-/-^ mice demonstrated that DGAT1 was required for hepatic steatosis caused by a high-fat diet or fasting, both of which promote hepatic uptake of exogenous FAs, but not for hepatic steatosis caused by upregulation of endogenous de novo FA synthesis^65^. As a result, the low DGAT1 expression observed in the presence of Ex-4 is most likely a response to reduced FAs uptake rather than reduced de novo lipogenesis, ruling out a role for reduced de novo lipogenesis in the Ex-4-induced steatosis improvement.

A decrease in ACC expression stimulates lipid β-oxidation by reducing the production of the β-oxidation inhibitor malonyl-CoA^66^. Thus, an increased β-oxidation might explain the improved steatosis we observe in the presence of Ex-4. Nevertheless, this possibility is ruled out by the fact that Ex-4 decreases the expression of CPT1, the rate-limiting enzyme for mitochondrial β-oxidation^67^.

OA treatment significantly increases the expression of ApoB, an essential protein for the assembly and secretion of TG-rich ApoB-containing lipoproteins, such as VLDL^68^. This increase in ApoB expression likely reflects a compensatory mechanism to enhance the secretion of VLDL and hence reduce the content of TGs. Nonetheless, Ex-4 significantly reduces the OA-induced upregulation of ApoB (Fig. 2C). This finding is in line with a previous study, which reported that continuous administration of fat diet-fed APOE*3-Leiden transgenic mice with Ex-4 or CNTO3649, a GLP-1 peptide analog, results in reduced hepatic TGs, cholesterol, and phospholipids in addition to down-regulation of ApoB expression^69^. Thereby, this observation excludes the significant contribution of enhanced lipid export to the Ex-4-induced steatosis reduction. Interestingly, the Ex-4-induced reduction of ApoB expression was blunted by the silencing of β-catenin (Fig. 3G,H), indicating its dependency on β-catenin signaling.

The transcription factor FOXA1 is among the most effective activators of human FABP1^70^. We show that the presence of Ex-4 significantly reduces the FOXA1 expression relative to OA alone (Fig. 2C), which may, in turn, decrease FABP1 expression. Interestingly, FOXA1 is downregulated in liver samples from humans and rats with simple steatosis^71^, probably as a feedback mechanism to reduce FAs uptake by FABP1. Furthermore, FOXA1 promotes fatty acid breakdown by inducing peroxisomal fatty acid b-oxidation^71^. Nonetheless, given the reduced FOXA1 expression induced by Ex-4 in our study, it is unlikely that the observed Ex-4-induced TG content reduction is due to the stimulation of peroxisomal fatty acid -oxidation. Ex-4 induces a significant downregulation of FOXA1 (Fig. 2C) compared to steatotic cells. However, this downregulation is abrogated upon silencing of β-catenin (Fig. 3G,H), suggesting a role of the Wnt/β-catenin pathway in this process.

**Figure 5:**
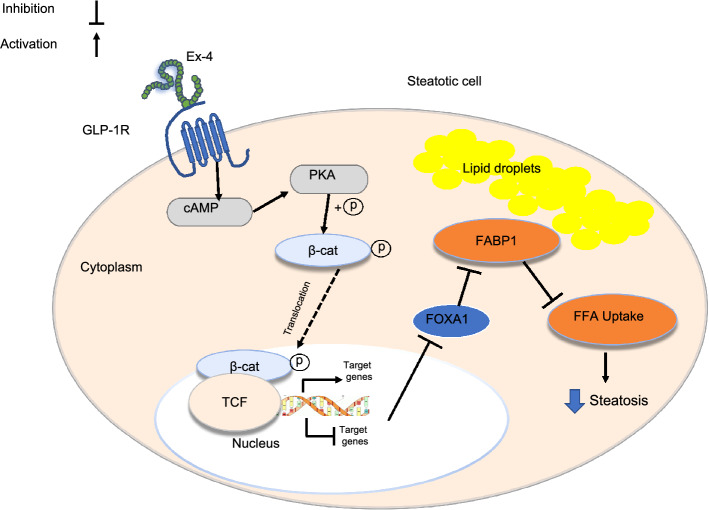
Proposed simplified signaling mechanism for Exendin-4-induced steatosis improvement. Exendin-4 action is mediated by directly binding to the Glucagon-Like Peptide-1 Receptor (GLP-1R) on the plasma membrane. The activation of the GLP-1R, which is coupled positively to the adenylyl cyclase (AC) system, stimulates AC and increases intracellular cAMP and activation of protein kinase A (PKA). The PKA phosphorylates and activates β-catenin, which is subsequently translocated to the nucleus. Nuclear β-catenin then binds to T-cell factor (Tcf) to form a bipartite transcription factor and facilitates the positive or negative modulation of the Tcf-dependent genes, leading, among other effects, to reduced expression of the transcription factor Forkhead Box A1 (FOXA1). The lower expression of FOXA1, one of the most effective activators of the human Fatty Acid Binding Protein 1 (FABP1), results in downregulation of FABP1, which leads to reduced uptake and transport of fatty acids and ultimately decreased steatosis.

The involvement of the β-catenin signaling in the Ex-4-induced improvement in hepatic steatosis was suggested previously by Seo and coworkers^37^ who showed that the β-catenin inhibitor IWR-1 abrogates the protective effect of Ex-4 against palmitate-induced steatosis. Our results also indicate the potential involvement of the β-catenin signaling pathway by showing the impact of Ex-4 on the expression of nuclear transcription factors SREBP-1, a key regulator of lipid metabolism in the liver^72^, and TCF4, a central transcription factor in the β-catenin pathway, when β-catenin is silenced. Hence, after β-catenin knockdown, OA treatment significantly upregulates both SREBP-1 and TCF4 (Fig. 3D–F). However, the presence of Ex-4 drastically reduces this upregulation. Interestingly, in the context of Wnt/β-catenin signaling-dependent liver tumorigenesis, it was suggested that TCF4 might act in concert with the FOXA factors to regulate hepatocellular carcinoma-specific Wnt target gene expression^73^. Therefore, GLP-1R stimulation may activate the β-catenin pathway, which may result in a concerted action by TCF4 and FOXA1 to regulate the expression of FABP1 and hence prevent the lipid accumulation induced by OA (Fig. 5). It is worth noting that FABP1was suggested as a critical driver gene in hepatitis B X-protein-induced hepatic lipid accumulation^74^. However, further investigations are warranted to decipher the complete mechanism underlying the protective effect of GLP1R agonists against hepatic steatosis.

In conclusion, the present study proposes that the direct activation of GLP-1R by Ex-4 reduces OA-induced steatosis in HepG2 cells by stimulating the Wnt/β-catenin signaling pathway, which reduces FOXA1 expression. FOXA1 downregulation, in turn, reduces FABP1 expression, which ultimately leads to a decrease in FFAs uptake. Targeting FABP1 expression in the liver could be beneficial as a medical treatment for fatty liver disease.

## Supplementary Information

Supplementary Information 1.

Supplementary Information 2.

Supplementary Information 3.

Supplementary Information 4.

Supplementary Information 5.

Supplementary Information 6.

Supplementary Information 7.

Supplementary Information 8.

Supplementary Information 9.

Supplementary Information 10.

Supplementary Information 11.

Supplementary Information 12.
