# Supplementary figures and images for: Exendin-4 alleviates steatosis in an in vitro cell model by lowering FABP1 and FOXA1 expression via the Wnt/-catenin signaling pathway
Source: Sci Rep. 2022 Feb 9;12:2226. doi: 10.1038/s41598-022-06143-5 (PMC8828858; doi:10.1038/s41598-022-06143-5)

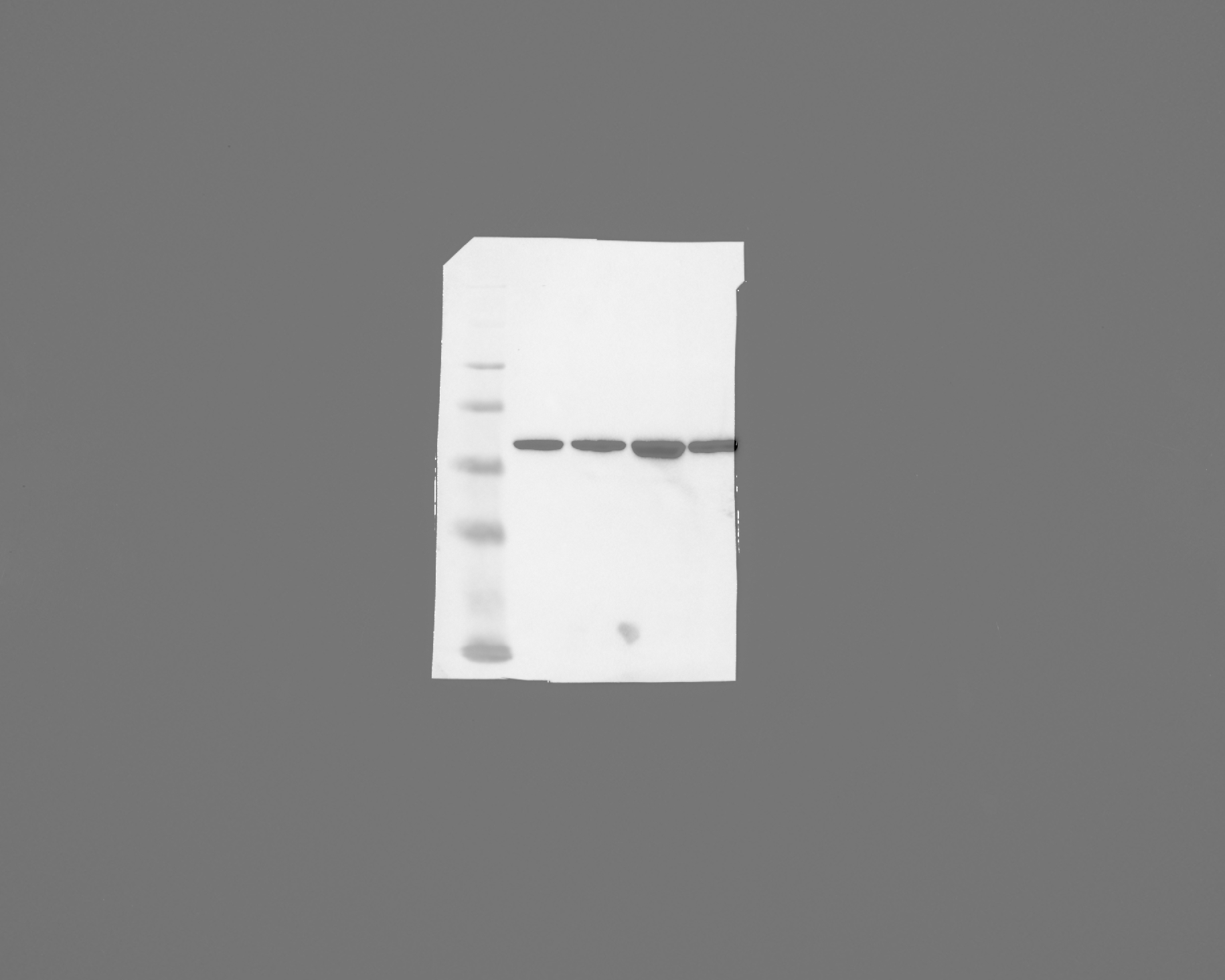

Supplement: Supplementary file 1 — Supplementary Information 1. [file 41598_2022_6143_MOESM1_ESM.jpg]

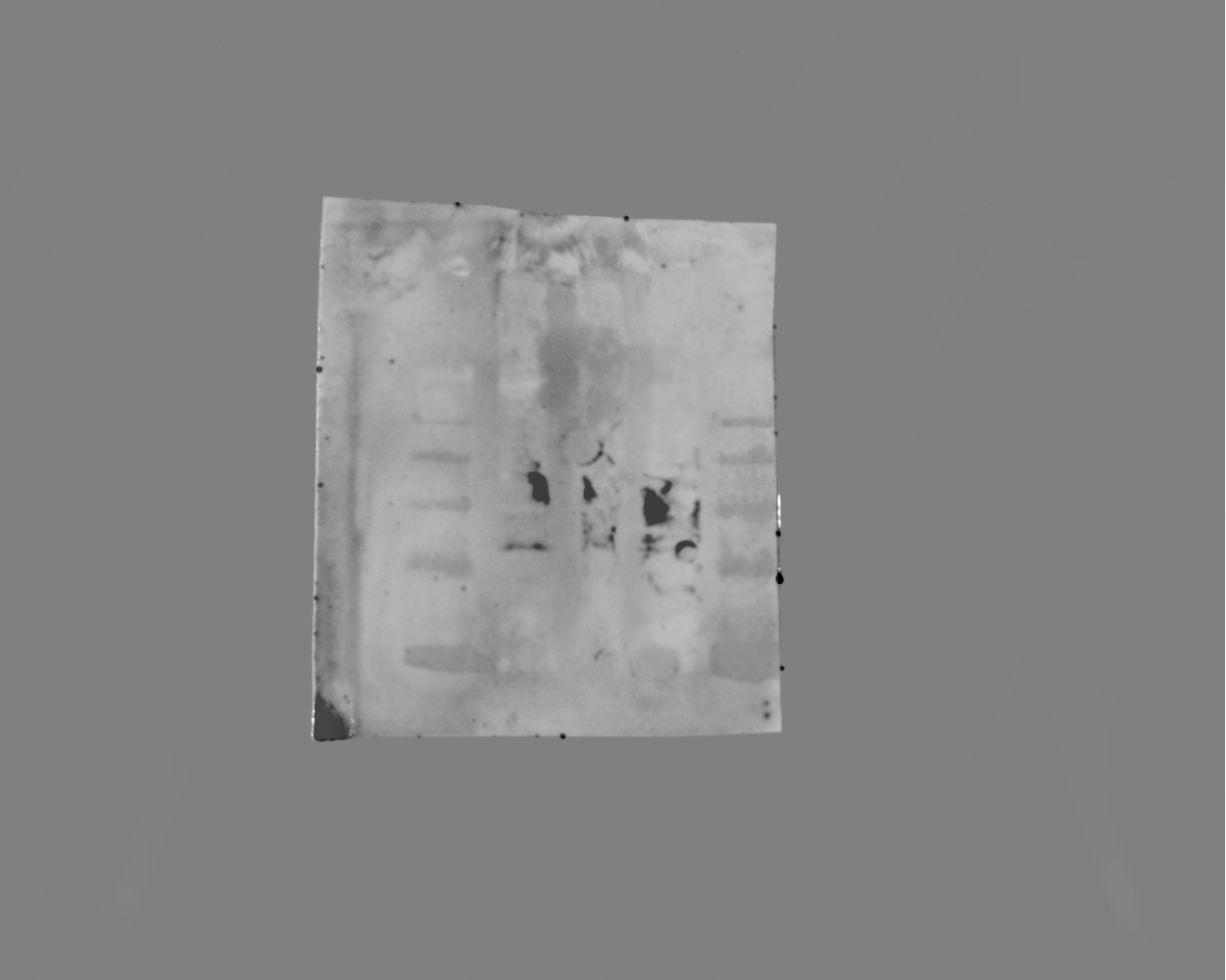

Supplement: Supplementary file 2 — Supplementary Information 2. [file 41598_2022_6143_MOESM2_ESM.jpg]

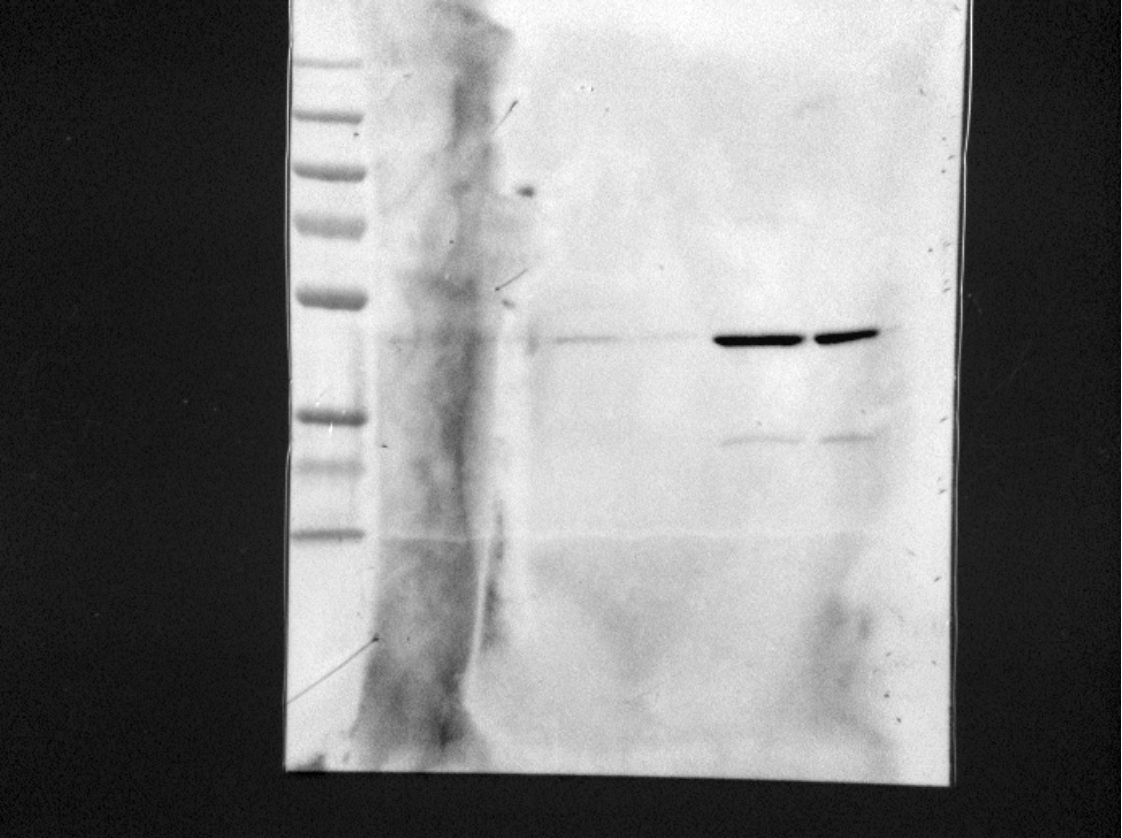

Supplement: Supplementary file 3 — Supplementary Information 3. [file 41598_2022_6143_MOESM3_ESM.tif]

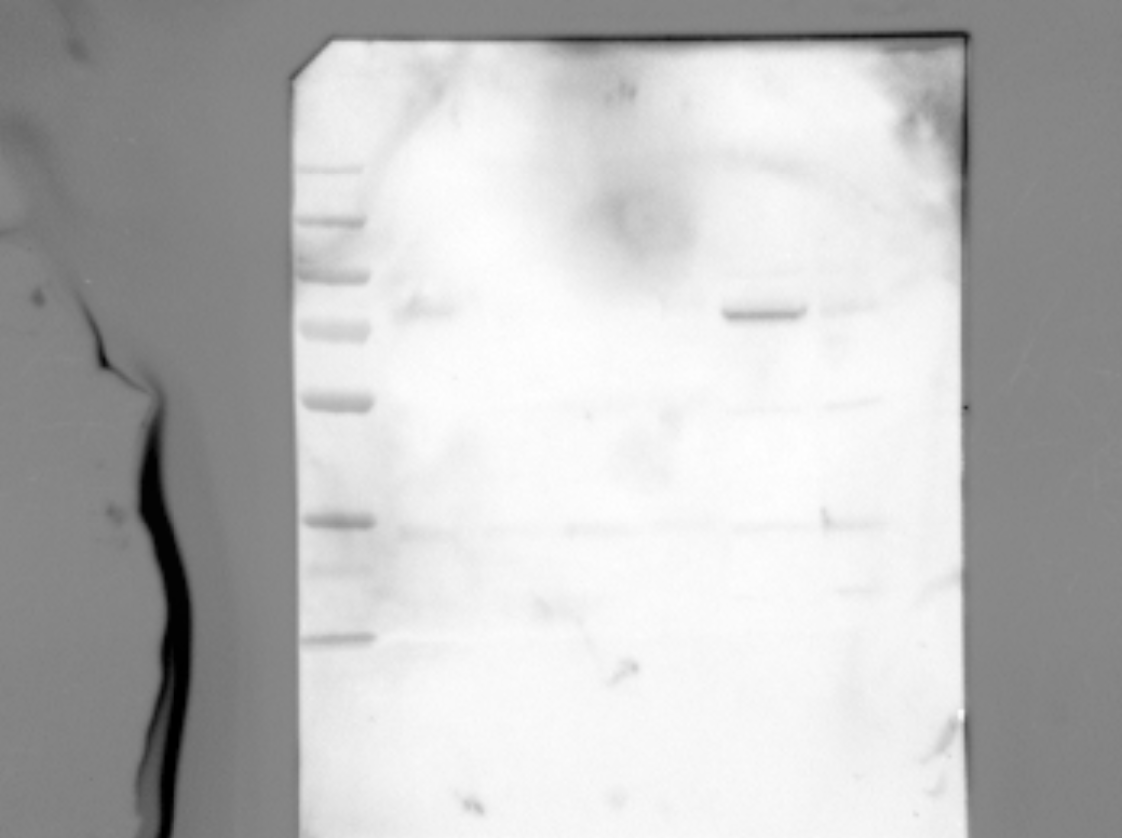

Supplement: Supplementary file 4 — Supplementary Information 4. [file 41598_2022_6143_MOESM4_ESM.tif]

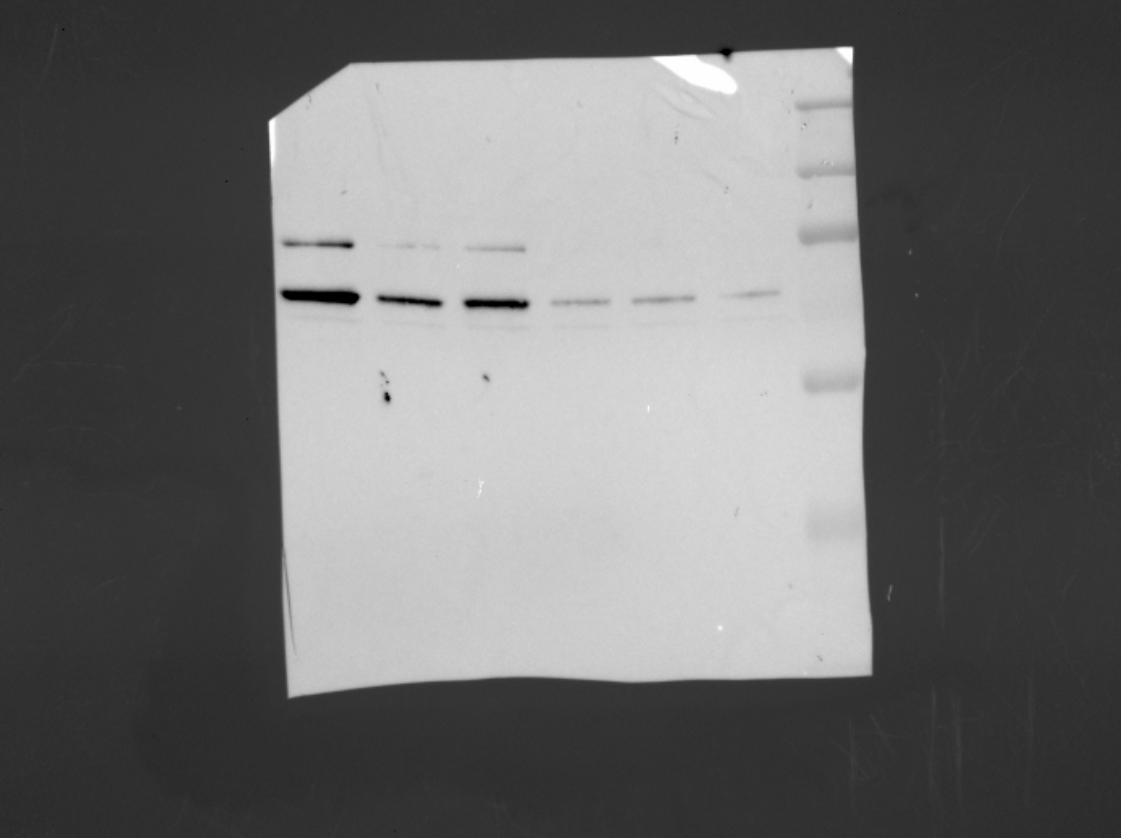

Supplement: Supplementary file 5 — Supplementary Information 5. [file 41598_2022_6143_MOESM5_ESM.tif]

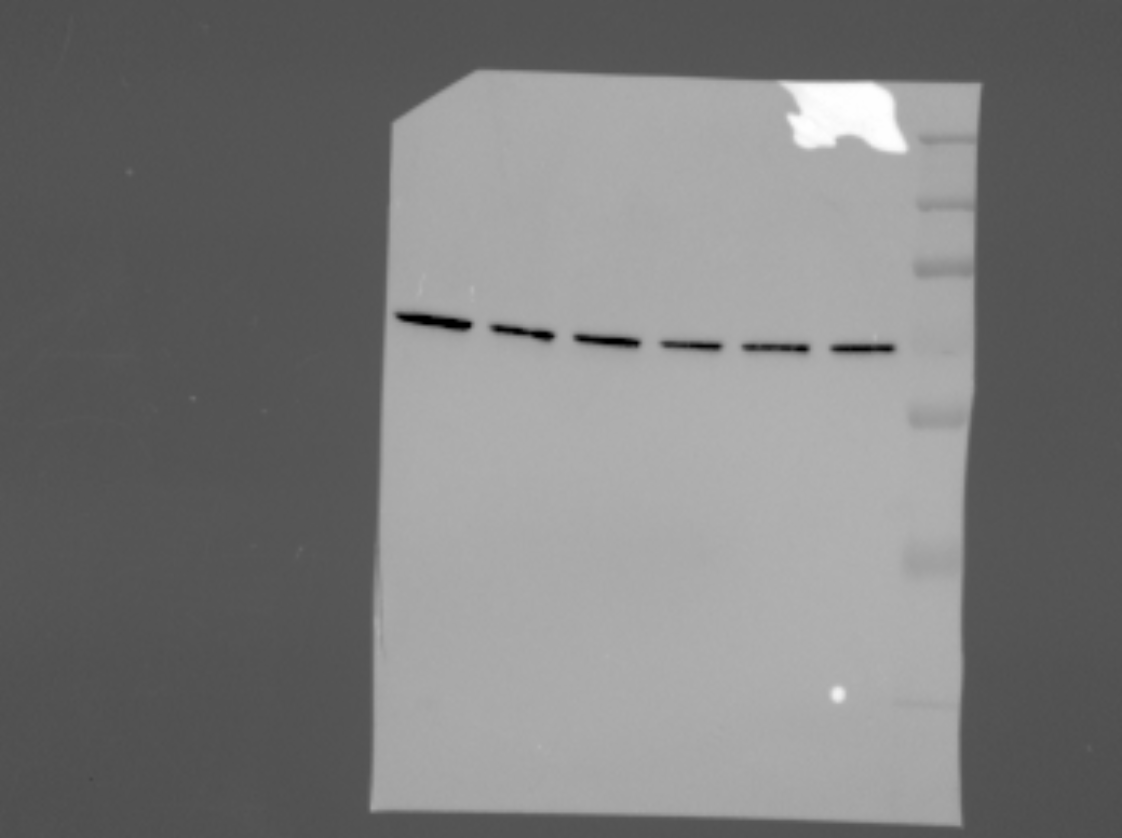

Supplement: Supplementary file 6 — Supplementary Information 6. [file 41598_2022_6143_MOESM6_ESM.tif]

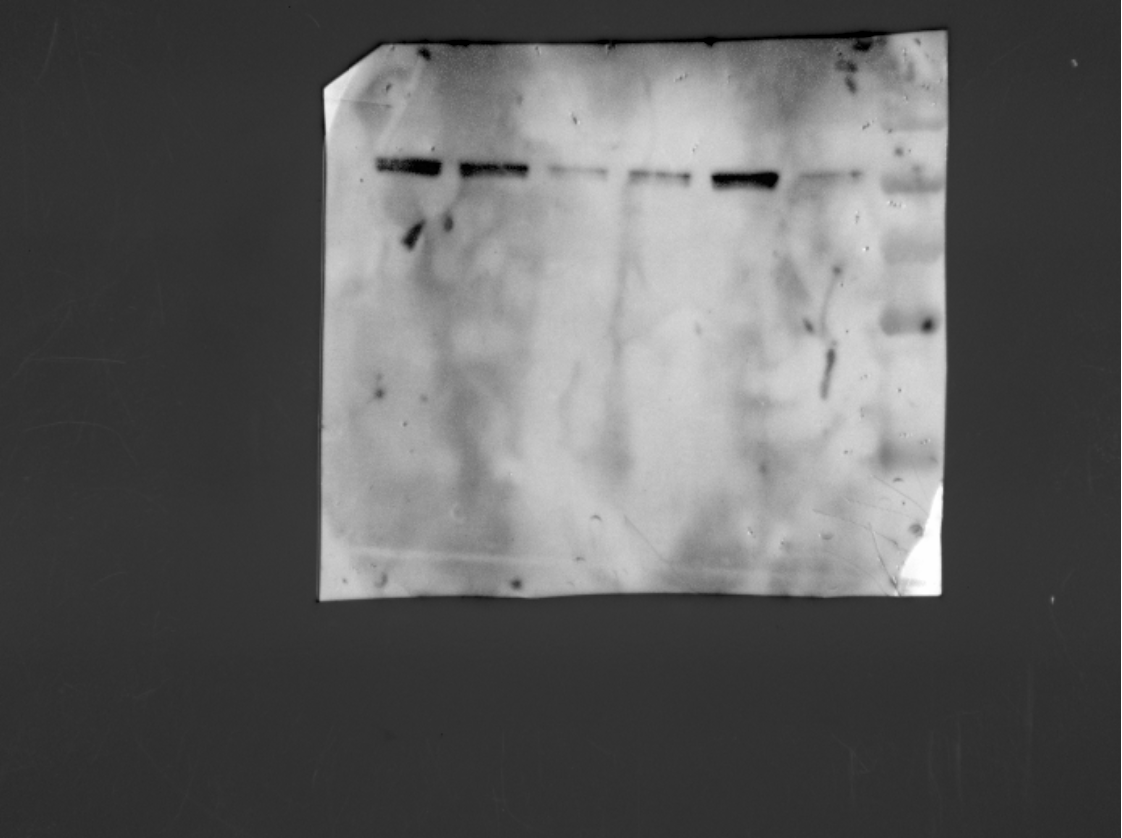

Supplement: Supplementary file 7 — Supplementary Information 7. [file 41598_2022_6143_MOESM7_ESM.tif]

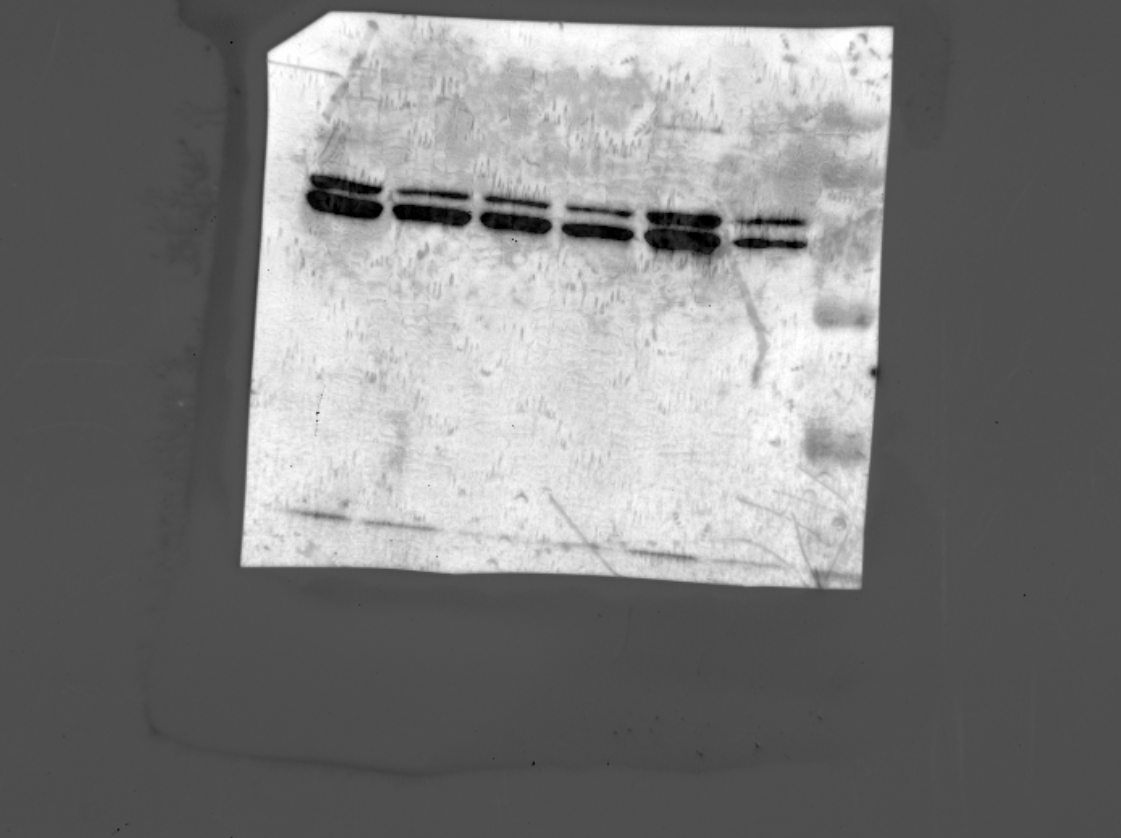

Supplement: Supplementary file 8 — Supplementary Information 8. [file 41598_2022_6143_MOESM8_ESM.tif]

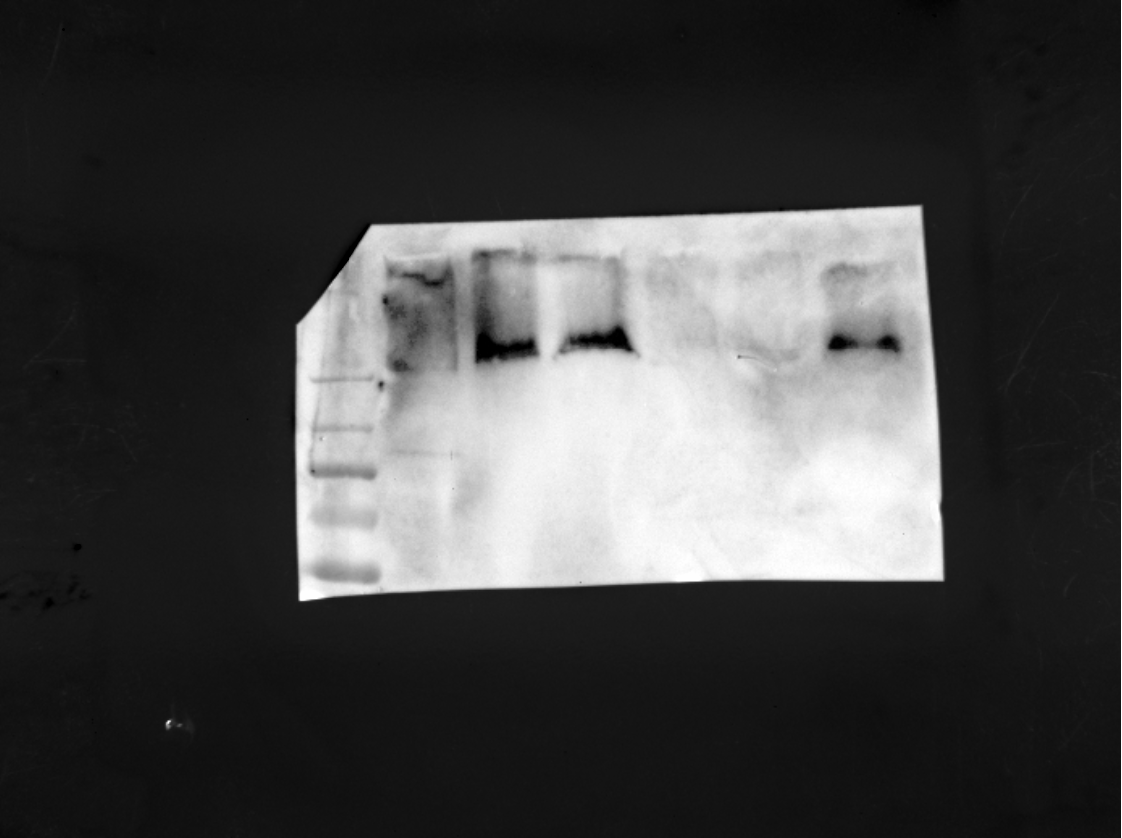

Supplement: Supplementary file 9 — Supplementary Information 9. [file 41598_2022_6143_MOESM9_ESM.tif]

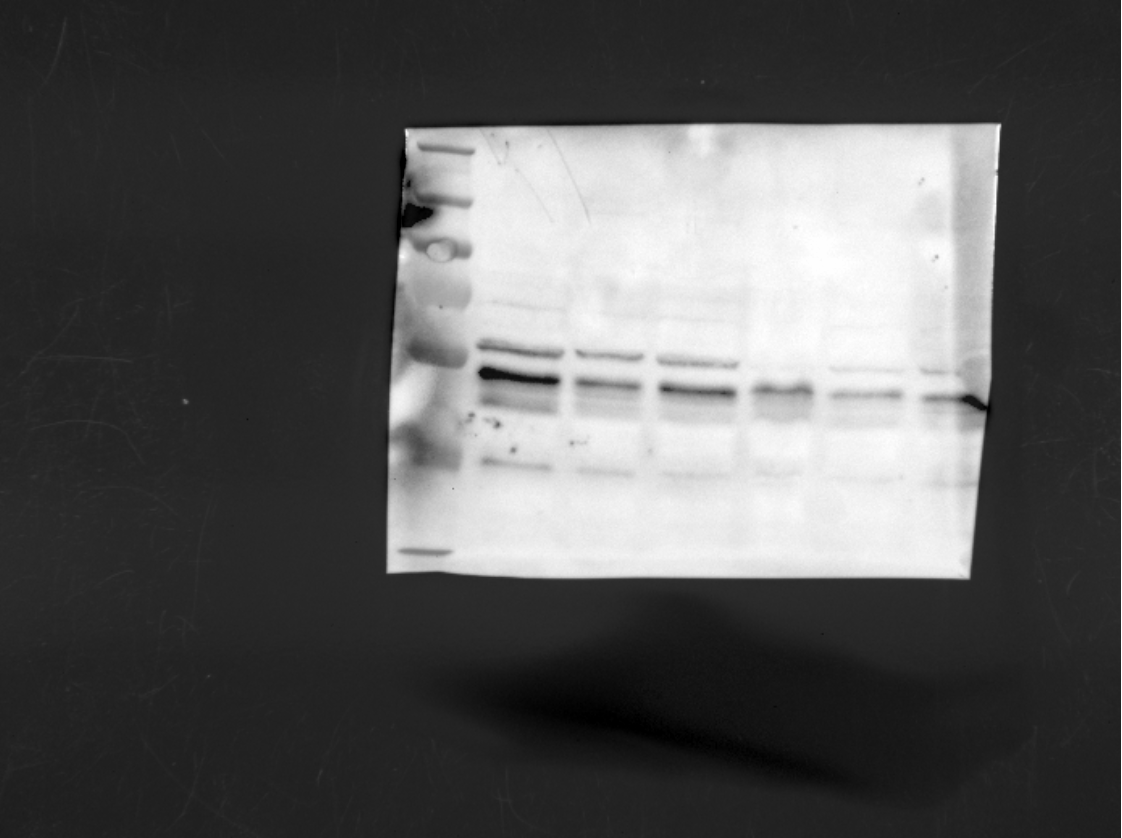

Supplement: Supplementary file 10 — Supplementary Information 10. [file 41598_2022_6143_MOESM10_ESM.tif]

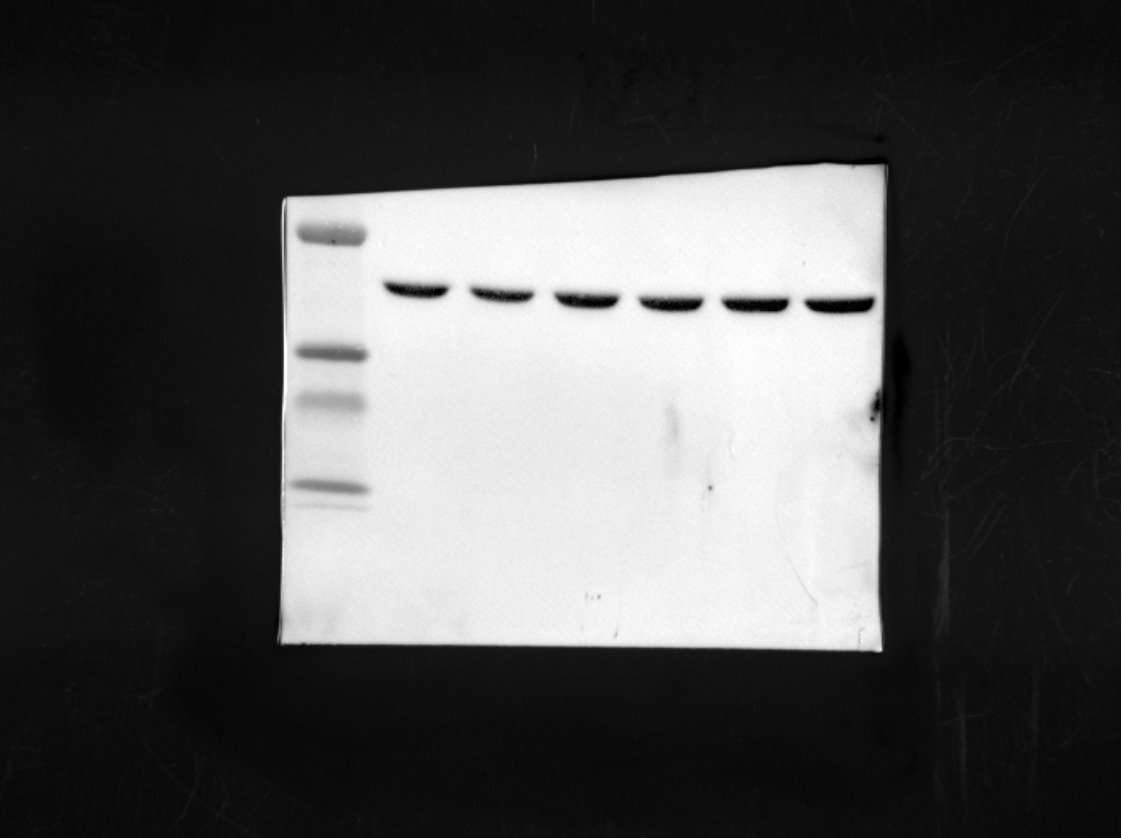

Supplement: Supplementary file 12 — Supplementary Information 12. [file 41598_2022_6143_MOESM12_ESM.tif]
