# Supplementary material for: Exendin-4 alleviates steatosis in an in vitro cell model by lowering FABP1 and FOXA1 expression via the Wnt/-catenin signaling pathway
Source: Sci Rep. 2022 Feb 9;12:2226. doi: 10.1038/s41598-022-06143-5 (PMC8828858; doi:10.1038/s41598-022-06143-5)
Supplement: Supplementary file 11 — Supplementary Information 11. [file 41598_2022_6143_MOESM11_ESM.pptx]

## Slide 1
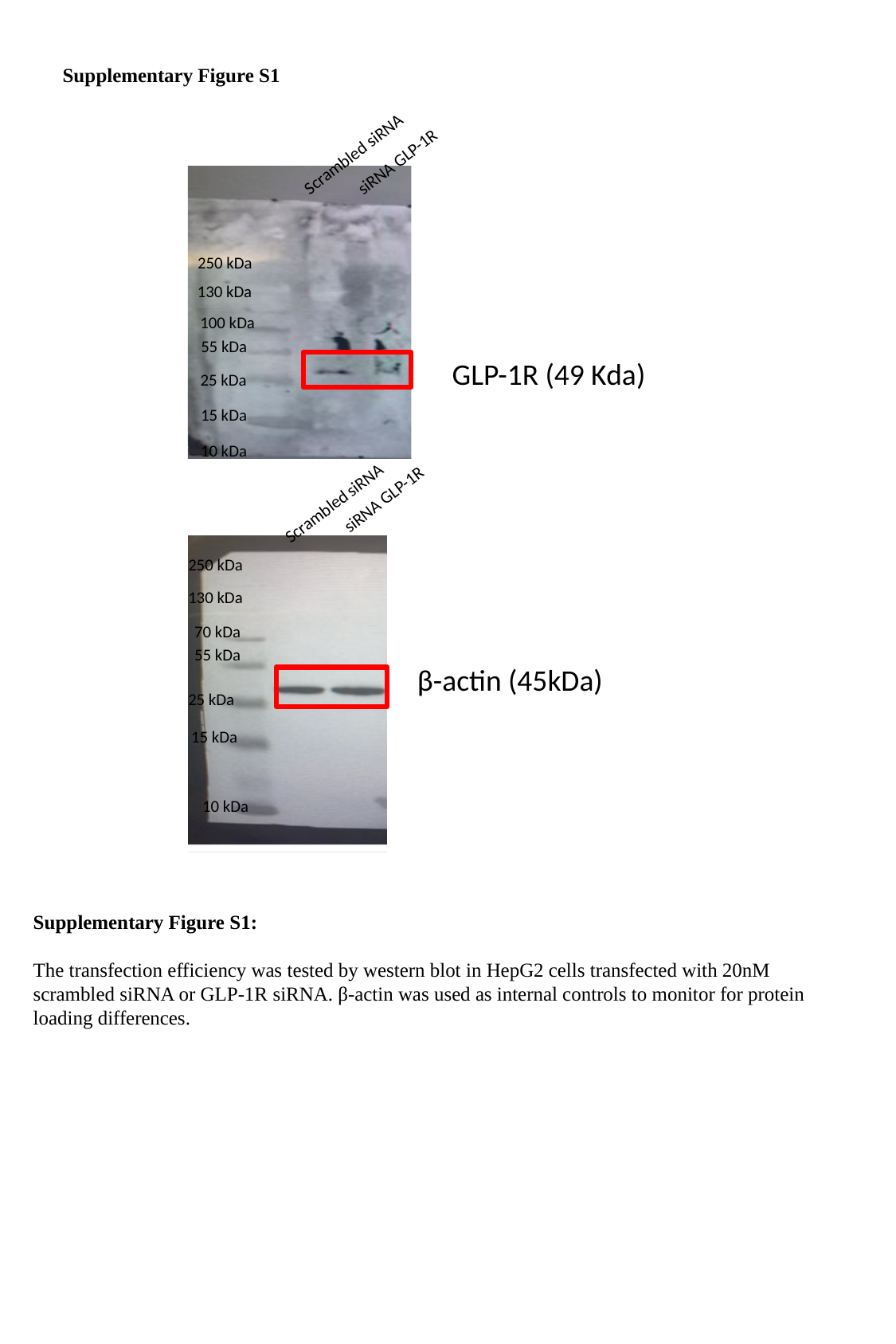

Supplementary Figure S1
Scrambled siRNA
GLP-1R (49 Kda)
siRNA GLP-1R
Scrambled siRNA
siRNA GLP-1R
250 kDa
130 kDa
100 kDa
55 kDa
25 kDa
15 kDa
10 kDa
250 kDa
130 kDa
70 kDa
55 kDa
β-actin (45kDa)
25 kDa
15 kDa
10 kDa
Supplementary Figure S1:
The transfection efficiency was tested by western blot in HepG2 cells transfected with 20nM scrambled siRNA or GLP-1R siRNA. β-actin was used as internal controls to monitor for protein loading differences.

## Slide 2
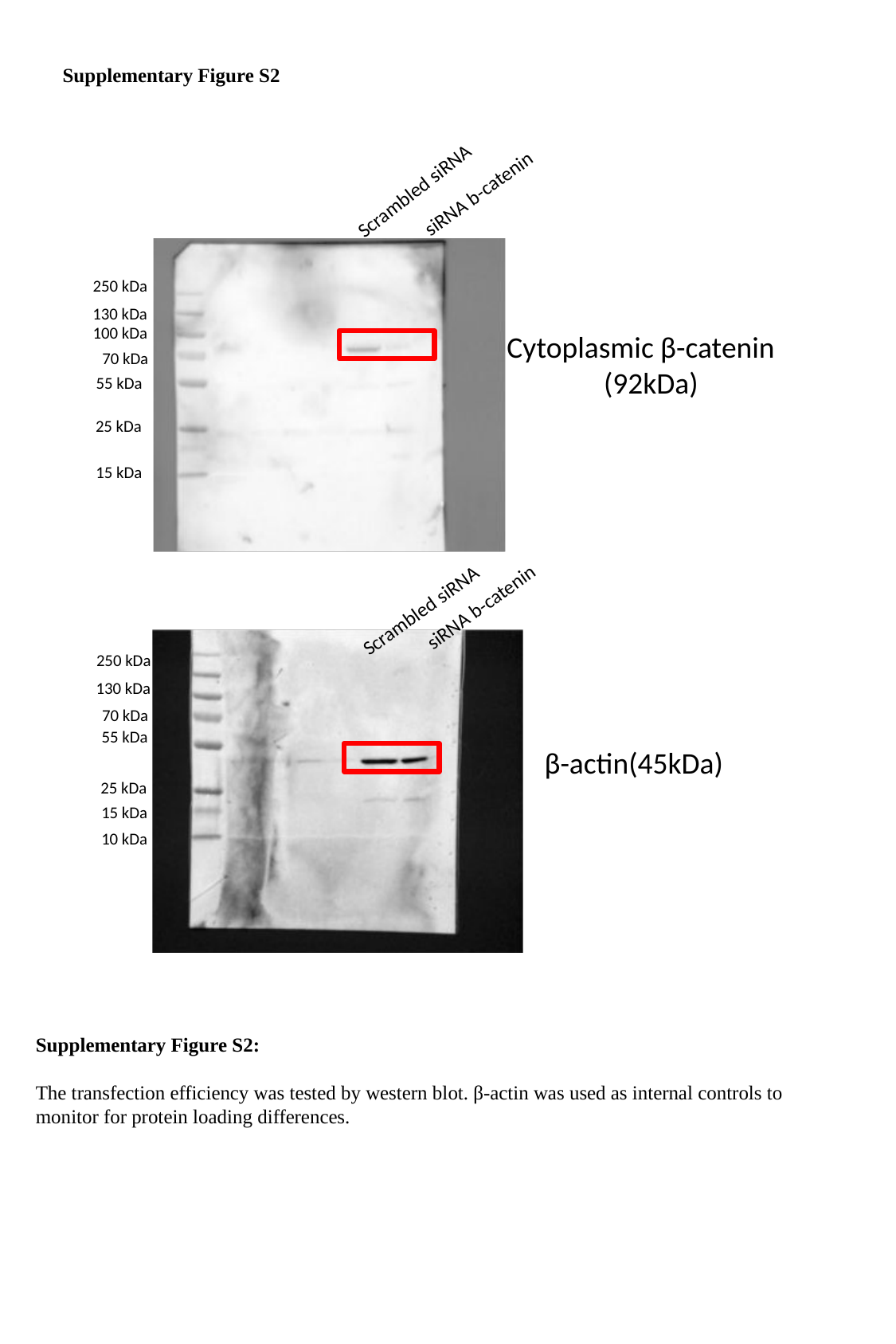

Supplementary Figure S2
Scrambled siRNA
siRNA b-catenin
Cytoplasmic β-catenin
(92kDa)
250 kDa
130 kDa
100 kDa
70 kDa
55 kDa
25 kDa
15 kDa
siRNA b-catenin
Scrambled siRNA
250 kDa
130 kDa
70 kDa
55 kDa
25 kDa
15 kDa
10 kDa
250 kDa
130 kDa
70 kDa
55 kDa
β-actin(45kDa)
25 kDa
15 kDav
10 kDav
Supplementary Figure S2:
The transfection efficiency was tested by western blot. β-actin was used as internal controls to monitor for protein loading differences.

## Slide 3
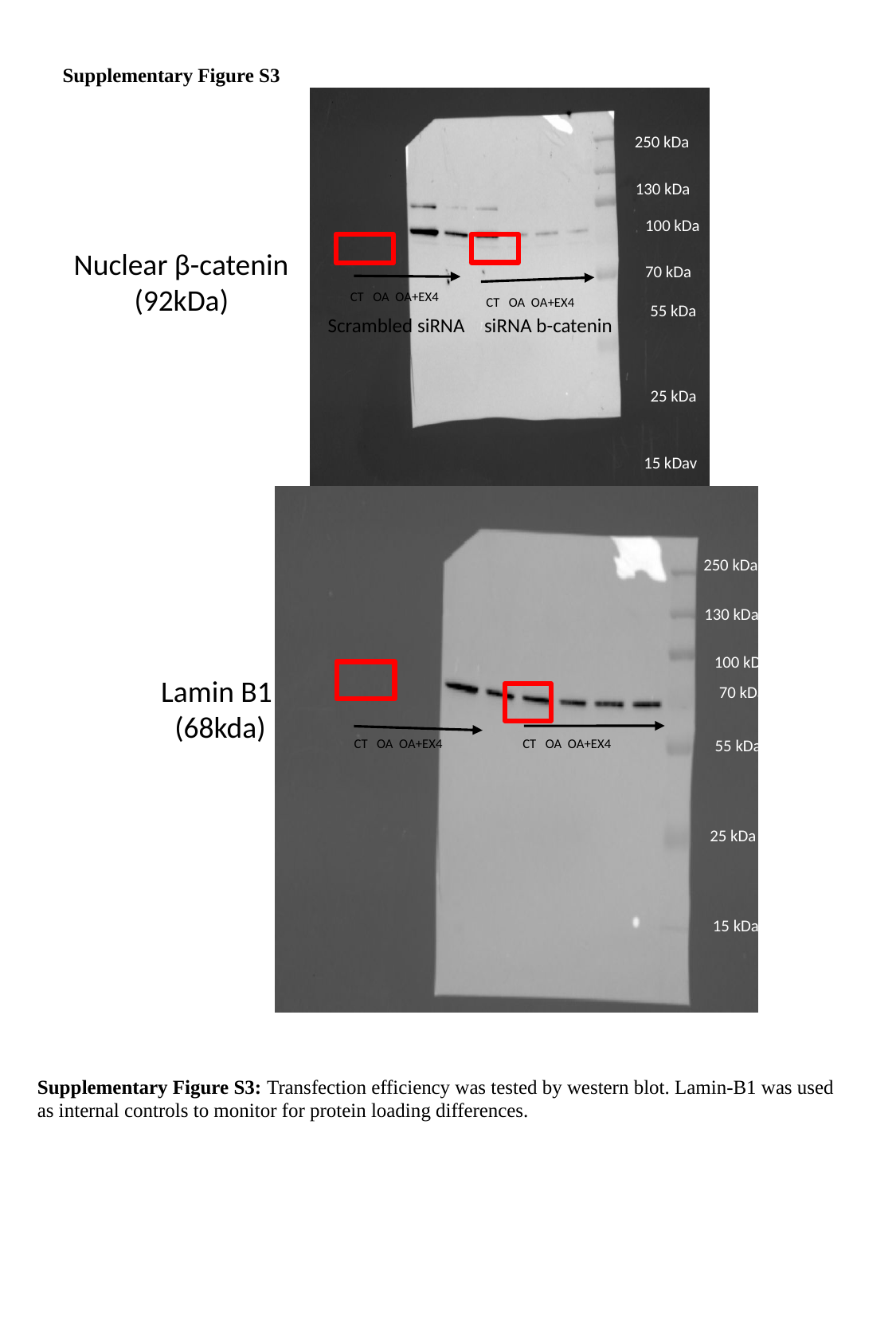

Supplementary Figure S3
250 kDa
130 kDa
100 kDa
Nuclear β-catenin
(92kDa)
70 kDa
CT OA OA+EX4
CT OA OA+EX4
55 kDa
siRNA b-catenin
Scrambled siRNA
25 kDa
15 kDav
250 kDa
130 kDa
100 kDa
Lamin B1
(68kda)
70 kDa
CT OA OA+EX4
55 kDa
CT OA OA+EX4
25 kDa
15 kDa
Supplementary Figure S3: Transfection efficiency was tested by western blot. Lamin-B1 was used as internal controls to monitor for protein loading differences.

## Slide 4
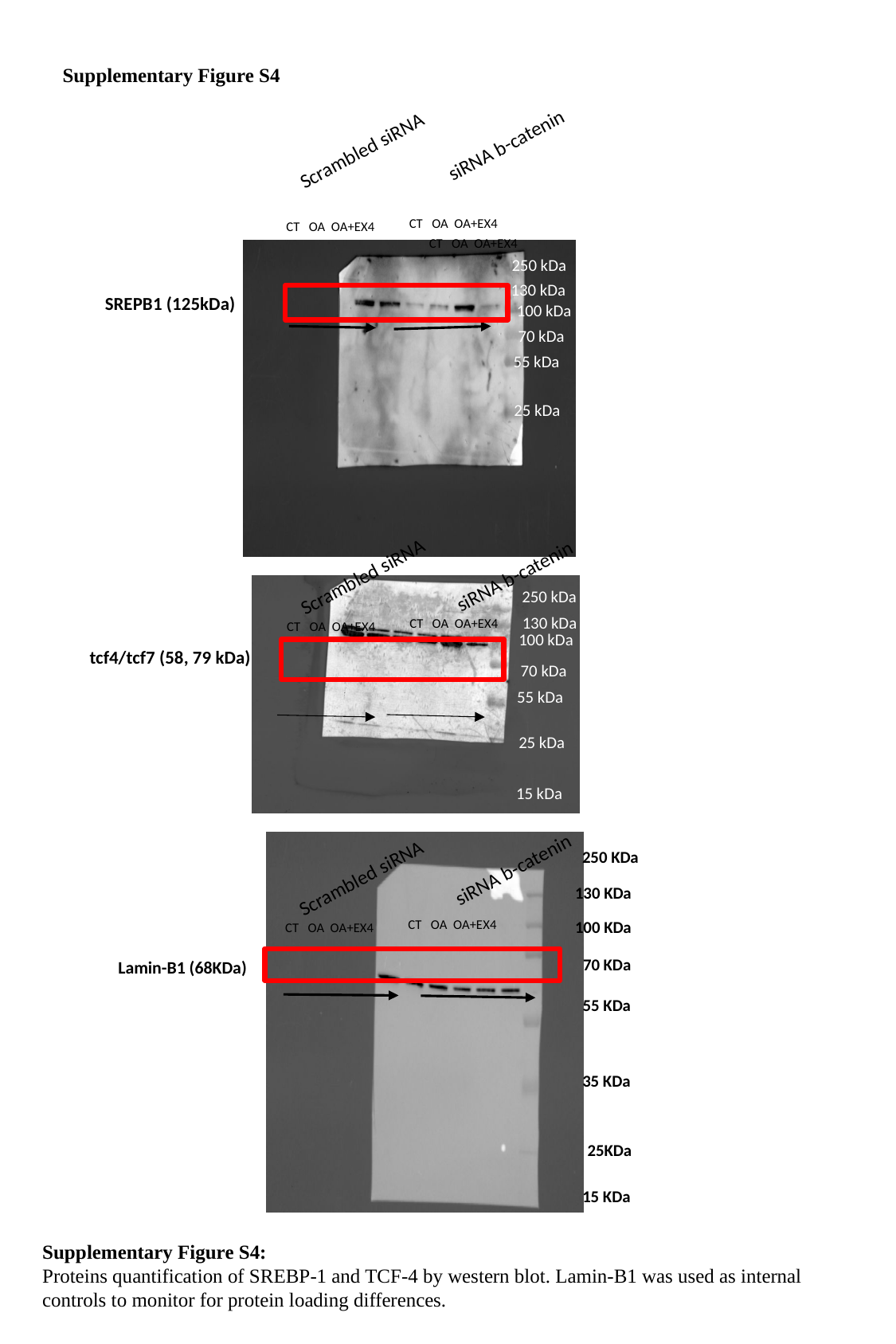

Supplementary Figure S4
siRNA b-catenin
Scrambled siRNA
CT OA OA+EX4
CT OA OA+EX4
CT OA OA+EX4
SREPB1 (125kDa)
250 kDa
130 kDa
100 kDa
70 kDa
55 kDa
25 kDa
B
C
siRNA b-catenin
Scrambled siRNA
tcf4/tcf7 (58, 79 kDa)
250 kDa
130 kDa
100 kDa
70 kDa
55 kDa
25 kDa
CT OA OA+EX4
CT OA OA+EX4
15 kDa
250 KDa
130 KDa
100 KDa
70 KDa
55 KDa
35 KDa
25KDa
15 KDa
Lamin-B1 (68KDa)
siRNA b-catenin
Scrambled siRNA
CT OA OA+EX4
CT OA OA+EX4
Supplementary Figure S4:
Proteins quantification of SREBP-1 and TCF-4 by western blot. Lamin-B1 was used as internal controls to monitor for protein loading differences.

## Slide 5
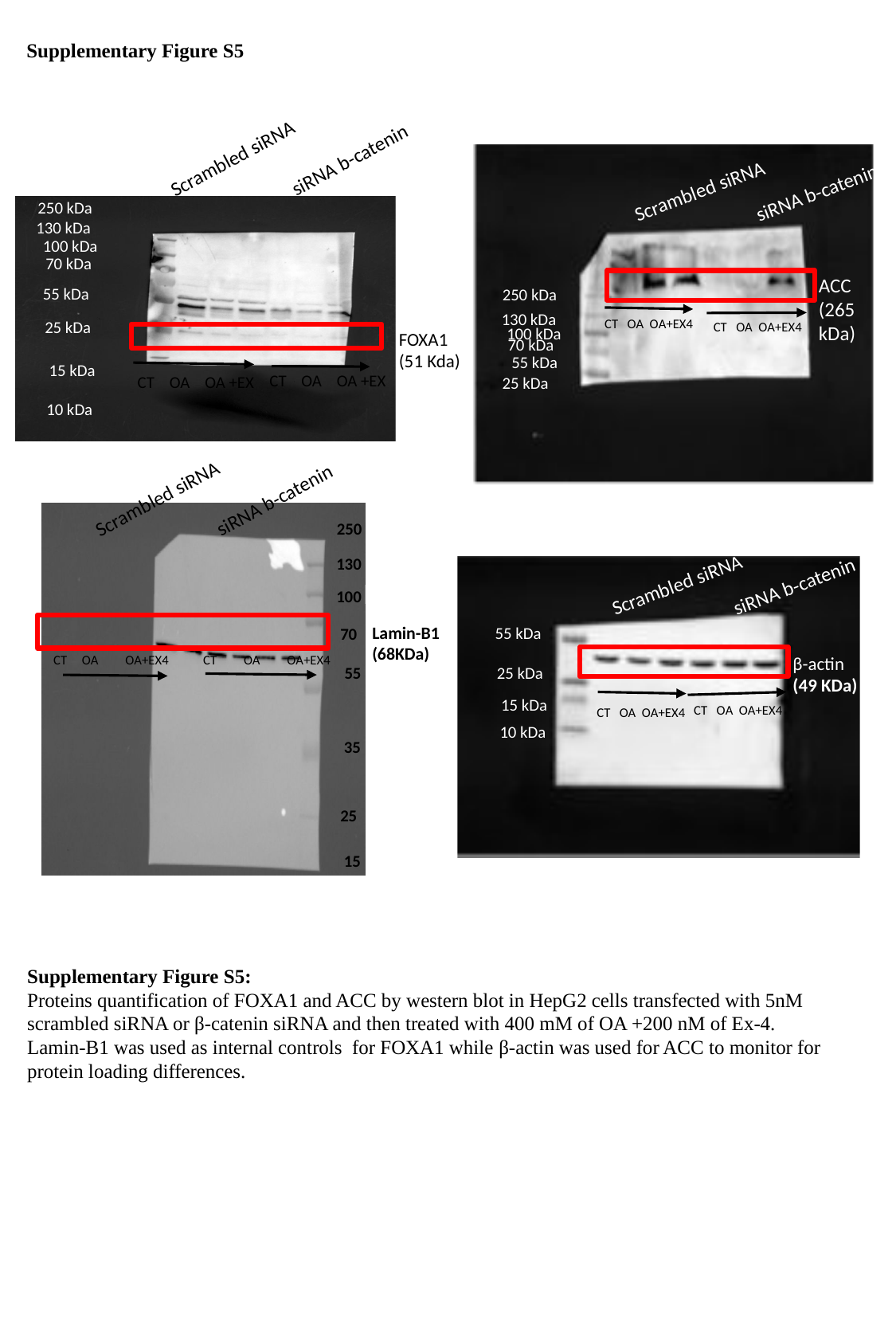

Supplementary Figure S5
Scrambled siRNA
siRNA b-catenin
ACC
(265 kDa)
CT OA OA+EX4
CT OA OA+EX4
CT OA OA+EX4
CT OA OA+EX4
Scrambled siRNA
siRNA b-catenin
250 kDa
130 kDa
100 kDa
70 kDa
55 kDa
25 kDa
15 kDa
CT OA OA +EX
10 kDa
FOXA1 (51 Kda)
250 kDa
130 kDa
100 kDa
70 kDa
55 kDa
CT OA OA +EX
25 kDa
Scrambled siRNA
siRNA b-catenin
250
130
100
70
55
35
25
15
Lamin-B1 (68KDa)
Scrambled siRNA
siRNA b-catenin
D
55 kDa
CT OA OA+EX4
CT OA OA+EX4
β-actin (49 KDa)
25 kDa
15 kDa
10 kDa
Supplementary Figure S5:
Proteins quantification of FOXA1 and ACC by western blot in HepG2 cells transfected with 5nM scrambled siRNA or β-catenin siRNA and then treated with 400 mM of OA +200 nM of Ex-4. Lamin-B1 was used as internal controls for FOXA1 while β-actin was used for ACC to monitor for protein loading differences.
